# Diet, Immunity, and Microbiota Interactions: An Integrative Analysis of the Intestine Transcriptional Response and Microbiota Modulation in Gilthead Seabream (*Sparus aurata*) Fed an Essential Oils-Based Functional Diet

**DOI:** 10.3389/fimmu.2021.625297

**Published:** 2021-03-04

**Authors:** Joana P. Firmino, Eva Vallejos-Vidal, M. Carmen Balebona, Yuliaxis Ramayo-Caldas, Isabel M. Cerezo, Ricardo Salomón, Lluis Tort, Alicia Estevez, Miguel Ángel Moriñigo, Felipe E. Reyes-López, Enric Gisbert

**Affiliations:** ^1^IRTA, Centre de Sant Carles de la Ràpita (IRTA-SCR), Aquaculture Program, Sant Carles de la Ràpita, Spain; ^2^TECNOVIT–FARMFAES, S.L. Alforja, Spain; ^3^Ph.D. Program in Aquaculture, Universitat Autònoma de Barcelona, Barcelona, Spain; ^4^Departamento de Biología, Facultad de Química y Biología, Centro de Biotecnología Acuícola, Universidad de Santiago de Chile, Santiago, Chile; ^5^Department of Microbiology, Faculty of Science, University of Malaga, Málaga, Spain; ^6^Animal Breeding and Genetics Program, Institute of Agrifood Research and Technology, Torre Marimon, Caldes de Montbui, Spain; ^7^Department of Cell Biology, Physiology and Immunology, Universitat Autònoma de Barcelona, Barcelona, Spain; ^8^Facultad de Medicina Veterinaria y Agronomía, Universidad de Las Américas, Santiago, Chile; ^9^Consorcio Tecnológico de Sanidad Acuícola, Ictio Biotechnologies S. A., Santiago, Chile

**Keywords:** gut-associated lymphoid tissue, microbiota, additive, functional feed, aquaculture, oral immmunization, teleost, gut immune crosstalk

## Abstract

Essential oils (EOs) are promising alternatives to chemotherapeutics in animal production due to their immunostimulant, antimicrobial, and antioxidant properties, without associated environmental or hazardous side effects. In the present study, the modulation of the transcriptional immune response (microarray analysis) and microbiota [16S Ribosomal RNA (rRNA) sequencing] in the intestine of the euryhaline fish gilthead seabream (*Sparus aurata*) fed a dietary supplementation of garlic, carvacrol, and thymol EOs was evaluated. The transcriptomic functional analysis showed the regulation of genes related to processes of proteolysis and inflammatory modulation, immunity, transport and secretion, response to cyclic compounds, symbiosis, and RNA metabolism in fish fed the EOs-supplemented diet. Particularly, the activation of leukocytes, such as acidophilic granulocytes, was suggested to be the primary actors of the innate immune response promoted by the tested functional feed additive in the gut. Fish growth performance and gut microbiota alpha diversity indices were not affected, while dietary EOs promoted alterations in bacterial abundances in terms of phylum, class, and genus. Subtle, but significant alterations in microbiota composition, such as the decrease in *Bacteroidia* and *Clostridia* classes, were suggested to participate in the modulation of the intestine transcriptional immune profile observed in fish fed the EOs diet. Moreover, regarding microbiota functionality, increased bacterial sequences associated with glutathione and lipid metabolisms, among others, detected in fish fed the EOs supported the metabolic alterations suggested to potentially affect the observed immune-related transcriptional response. The overall results indicated that the tested dietary EOs may promote intestinal local immunity through the impact of the EOs on the host-microbial co-metabolism and consequent regulation of significant biological processes, evidencing the crosstalk between gut and microbiota in the inflammatory regulation upon administration of immunostimulant feed additives.

## Introduction

In the post-antibiotics era, concerns about the potential loss of productivity due to an increase of infectious diseases are a reality within the animal production sector. The production of aquatic protein through aquaculture is not an exception. Regarding the aquaculture industry, the increasing pathogen resistance to chemotherapeutic treatments and their use restrictions, along with the rising public awareness regarding food safety, environmental impact, and animal welfare, have encouraged the development of alternative dietary treatments, such as functional feeds (1). Similarly, to livestock, functional feed additives may benefit farmed fish through the regulation of the host metabolism, nutrient absorption, and enhancement of host performance. Furthermore, it may activate the immunity of the host either by direct stimulation of the innate immune system or through the sustenance of commensal microorganisms and inhibition of pathogens in the intestinal tract (2). These factors, either individually or combined, may have a profound impact on key performance indicators. For instance, fish farmers recognize the direct effect of health promotion through feed in their economic gain by the improvement of key performance indicators (i.e., improving somatic growth, reducing feed conversion rates, promoting host welfare, and diminishing morbidity, among others). The wide array of potential benefits derived from functional feed additives and nutraceuticals has focused the light on their study and validation in aquafeeds. Among functional feed additives, phytogenics consist of a heterogeneous group of plant-derived products widely used in animal nutrition. Essential oils (EOs), a blend of organic substances synthesized by aromatic plants during secondary metabolism, whose chemical composition may vary according to plant and environment characteristics and/or extraction procedures, are the most common class of phytogenics used in livestock nutrition as well as in aquafeeds. EOs have been increasingly studied as promising chemotherapeutic alternatives due to their antimicrobial, immunostimulant, antioxidant, anti-stress, and growth-promoting properties, without associated environmental or hazardous side effects. In addition, there is also evidence that EOs may exert a positive impact on the gut health of livestock, including aquaculture relevant fish species (3). Particularly, some studies have recently reported the advantageous outcomes of the dietary administration of garlic (4), carvacrol, and/or thymol (5) in the gut health of aquatic species, which suggest them as interesting phytogenic targets for aquafeed additive development.

During the last years, the concept of “gut health” has become a trending topic due to its significance on the nutrition, metabolism, immunity, pathogen control, welfare, behavior, and performance of the host. Despite the term not being well-defined in the literature and perhaps having a subjective definition, the key components of this term in animal production are: (i) the diet, (ii) the functional structure of the mucosal barrier, (iii) an effective digestion and absorption of nutrients, (iv) an appropriate and stable microbiota, and (v) an effective immunity (6). Similar to higher vertebrates, teleost fish have a specialized and sophisticated gut immune system, although a significant variation is observed in the gastrointestinal tract of different species according to their nutritional requirements (7). The intestinal mucosal layer (including the mucus and the epithelial cells) form an important physical and biochemical defense barrier against exogenous substances, such as bacteria, toxins, and allergens. It also participates in the local immune response through the recognition and processing of antigens, the recruitment of innate and adaptive immune cells, and the secretion of cytokines, chemokines, antimicrobial peptides, and mucins through the gut-associated lymphoid tissue (GALT) (2).

A key actor in gut integrity and functionality is the microbial community that colonizes it. In this sense, the gut commensal microbiota, besides protecting the host against pathogenic bacteria invasion, are able to modulate the gene expression of processes involved in the stimulation of epithelial proliferation, nutrient metabolism, and innate immune responses, promoting intestinal homeostasis (3). Under this context, any interference on the intestinal mucosal barrier integrity and/or the microbiota composition may impair gut condition and health, leading to disease-related dysbiosis (8). An increasing body of evidence supports the general assumption that feed additives can influence considerably the fish gut condition, affecting the intestinal epithelium, microbiota, and mucosal immunity (2). There is also evidence that fish innate and adaptive immune system may influence the regulation and composition of the gut microbiota and *vice versa*. Such interactions are not clearly deciphered so far, especially in lower vertebrates (8). In this way, functional genomics studies provide a wide range of applications that allows an increased and better understanding of the mechanisms underlying this symbiosis. Consequently, this information is of paramount importance for deciphering the mode of action of feed additives and their proper dietary administration.

Under this context, the present study aimed to evaluate the intestinal tissue transcriptional activity and microbiota responses in the gut of the euryhaline fish gilthead seabream (*Sparus aurata*) fed a functional feed additive consisting of a microencapsulated blend of garlic, carvacrol, and thymol EOs. Additionally, the authors also sought to provide new insights about the shared role of host-microbial co-metabolism in building-up a local immune response promoted by the tested feed additive.

## Materials and Methods

### Rearing Conditions

Gilthead seabream fry (body weight, BW = 5.0 ± 0.2 g; mean ± SD) were purchased from Piscicultura Marina Mediterránea S.L. (Andromeda Group, Valencia, Spain) and transported to the research facilities of the Institute of Agrifood Research and Technology (IRTA) in Sant Carles de La Ràpita (Tarragona, Spain). Fish were randomly distributed among six tanks (450 L capacity) connected to the IRTAmar® recirculation system (5–10% water replacement per day for compensating evaporation and siphoning losses) in order to keep water quality through UV, biological, and mechanical filtration. At the beginning of the trial, 150 juveniles (25 fish per tank; initial density = 2 kg m^−3^) were individually measured in body weight (BW, g) and standard length (SL, mm) to the nearest 0.1 g and 1 mm, respectively (BW = 40.3 ± 0.1 g; SL = 12.0 ± 0.2 mm). This assay took place under natural photoperiod, with daily monitoring of the water temperature (25.1 ± 1.5°C), oxygen (6.8 ± 1.7 mg/L; >80% saturation) (OXI330, Crison Instruments, Barcelona, Spain), and pH (7.5 ± 0.01) (pHmeter 507, Crison Instruments), whereas salinity (35‰) (MASTER-20 T; ATAGO Co. Ltd), ammonia (0.13 ± 0.1 mg ${\text{NH}}_{4}^{+}$/L), and nitrite (0.18 ± 0.1 mg ${\text{NO}}_{2}^{-}$/L) levels (HACH DR9000 Colorimeter, Hach®, Spain) were weekly controlled.

### Diets and Feeding Trial

Diets were manufactured by Sparos Lda. (Olhão, Portugal) as follows: main ingredients were ground (below 250 μm) in a micropulverizer hammer mill (SH1; Hosokawa Micron, B.V., Doetinchem, The Netherlands). Powder ingredients and oils were then mixed according to the target formulation in a paddle mixer (RM90; Mainca, S.L., Granollers, Spain). After extrusion (2 mm pellet size), all feed batches were dried in a convection oven (OP 750-UF; LTE Scientifics, Oldham, UK) for 4 h at 45°C.

A basal (control) diet was formulated with high levels of marine-derived protein sources to contain 46% crude protein, 18% crude fat, and 21.5 MJ/kg gross energy (Table 1) as described in Firmino et al. (9). The second experimental diet was the control diet supplemented with 0.5% of the functional additive composed of a blend of microencapsulated garlic, carvacrol, and thymol synthetic EOs (AROTEC-G®, TECNOVIT-FARMFAES, S.L., Spain). When dealing with feed additives, especially EOs, their proper and controlled administration is of special importance. Thus, encapsulation technology was used for EOs incorporation into the experimental diet in order to improve its bioavailability and efficacy, as well as the standardization of its dosing in order to avoid variability and discrepancies among studies (10).

**Table 1:** Formulation and proximate composition of the basal diet.

**Ingredients**	**Basal diet (%)**
Fishmeal 70 LT FF Skagen	20.0
Fishmeal CORPESCA Super Prime	10.0
CPSP 90	2.5
Squid meal	2.5
Soy protein concentrate (Soycomil)	5.0
Wheat gluten	5.0
Corn gluten	8.0
Korfeed 60	4.5
Soybean meal 48	8.0
Rapeseed meal	4.0
Sunflower meal	3.0
Wheat meal	7.0
Whole peas	2.5
Fish oil—COPPENS	9.0
Soybean oil	1.5
Rapeseed oil	2.5
Vitamin and mineral Premix PV01	2.0
Soy lecithin—powder	2.0
Antioxidant powder (Paramega)	0.4
Dicalcium phosphate	0.6
TOTAL	100.0
**Proximate composition, % in dry basis**	
Crude protein	46.2
Crude fat	18.4
Gross Energy	21.5

Diets were tested for 65 days in a feeding trial carried out in triplicate tanks. Fish were hand-fed two times per day at the daily rate of 3.0% of the stocked biomass, which approached apparent satiation. At the end of the trial, all the fish in each tank were netted, anesthetized (buffered 150 mg/L MS-222, Sigma-Aldrich, Spain), and measured for BW and SL. We calculated different performance parameters: specific growth rate (SGR; % BW/day) = 100 × (ln BWf – ln BWi)/days (where BWf and BWi represented the final and the initial body weights, respectively). Fulton's condition factor (K) = (BWf/SLf ^3^) × 100 (where SLf was the final SL). This is a morphometric index that estimates the body condition of fish, assuming that heavier fish of a given length are in better condition (11).

In addition, four fish were randomly selected from each tank, euthanized with an overdose of the abovementioned anesthetic, and their gut removed. For transcriptional analysis purposes, a small section of the mid-anterior intestine from each fish was dissected, placed in RNAlater™ (Invitrogen, Thermo Fisher Scientific, Lithuania), incubated overnight (4°C), and stored at −80°C until further RNA extraction. There is evidence that the mid-anterior section of the fish intestine has a specialized immunological functionality when compared with other intestinal sections (12). The remaining sections of the anterior and posterior intestine were frozen separately in dry ice and stored at −80°C for further microbiota analysis.

### Transcriptional Analysis

#### RNA Isolation and Quality Control

Gilthead seabream mid-anterior intestine samples were randomly selected per dietary treatment. Total RNA was extracted individually using the RNeasy® Mini Kit (Qiagen, Germany) and eluted (final volume = 35 μl) in nuclease-free water and treated with DNAse (DNA-free™ DNA Removal Kit; Invitrogen, Lithuania). Total RNA concentration and purity were quantified using a Nanodrop-2000® spectrophotometer (Thermo Scientific, USA) and stored at −80°C. Prior to hybridization with microarrays, RNA samples were checked for RNA integrity (Agilent 2100 Bioanalyzer; Agilent Technologies, Spain) and selected by the criteria of a RIN value >8.5. Three different pools of samples per dietary treatment were established (*n* = 4 fish each).

#### Microarrays

A transcriptional analysis for the intestine from both experimental groups was carried out using the Aquagenomics *S. aurata* oligonucleotide microarray v2.0 (4 × 44 K) (SAQ) platform. Detailed information and transcriptomic raw data are available at the Gene Expression Omnibus (GEO) public repository at the U.S. National Center for Biotechnology Information (NCBI), accession numbers GPL13442 and GSE159643, respectively. The sampling labeling, hybridization, washes, and scanning were performed as described before (9). For this purpose, a one-color RNA labeling was used (Agilent One-Color RNA Spike-In Kit; Agilent Technologies, USA). RNA from each sample pool (200 ng) was reverse-transcribed together with the RNA spike-in. Then, total RNA was used as a template for Cyanine-3 (Cy3) labeled cRNA synthesis and amplification with the Quick Amp Labeling Kit (Agilent Technologies). cRNA samples were purified using the RNeasy Micro Kit (Qiagen). Dye incorporation and cRNA yield were checked (NanoDrop ND-2000® spectrophotometer). Then, Cy3-labeled cRNA (1.5 mg) with specific activity > 6.0 pmol Cy3/mg cRNA was fragmented at 60°C for 30 min, and hybridized with the array in presence of a hybridization buffer (Gene Expression Hybridization Kit, Agilent Technologies) at 65°C for 17 h. For washes, microarrays were incubated with Gene Expression wash buffers and stabilization and drying solution according to the manufacturer's instructions (Agilent Technologies). Microarray slides were then scanned (Agilent G2505B Microarray Scanner System), and spot intensities and other quality control features were extracted (Agilent Feature Extraction software version 10.4.0.0).

#### Intestine Functional Analysis: The Search Tool for the Retrieval of Interacting Genes

The Search Tool for the Retrieval of Interacting Genes (STRING) public repository version 11.0 (https://string-db.org) was used to generate the transcripteractome that takes place in the intestine of fish fed the EOs-supplemented diet. A Protein–Protein interaction (PPI) Networks Functional Enrichment Analysis for all the differentially expressed genes (DEGs) was conducted with a high-confidence interaction score (0.9) using *Homo sapiens* as model organism. Gene ontology (GO) enrichment analysis (*p* < 0.05) was also assessed including all the DEGs obtained. To confirm gene orthologs and match gene acronyms between both *H. sapiens* and gilthead seabream species, protein BLAST was run, and the GeneCards (www.genecards.org) and UniProt (www.uniprot.org) databases were accessed as described in Firmino et al. (9).

### Intestinal Microbiota

#### DNA Extraction

Samples were thawed gradually on ice, and the intestinal contents were extracted by pressing toward the ends with a sterile object. After homogenizing the content, a sample (50 mg) was taken for DNA extraction following the protocol based on saline precipitation (13). DNA concentration was quantified fluorometrically with the Qubit™ dsDNA HS Assay Kit (Thermo Fisher Scientific, Waltham, MA, USA) and its purity and integrity assessed using a NanoDrop™ One UV-Vis Spectrophotometer WiFi (Thermo Scientific, USA) and through an agarose gel electrophoresis.

#### Amplicon Sequencing of 16S Ribosomal RNA and Sequence Data Processing

The 16S rRNA of samples were sequenced on an Illumina® MiSeq platform (Illumina, San Diego, CA, USA) with 2 × 300 bp paired-end sequencing in the Ultrasequencing Service of the Bioinnovation Center of University of Málaga (Málaga, Spain). Sequencing was carried out using the sense forward 5′ TCGTCGGCAGCGTCAGATGTGTATAAGAGACAGCCTACGGGNGGCWGCAG 3′ and 5′ GTCTCGTGGGCTCGGAGATGTGTATAAGAGACAGGACTACHVGGGTATCTAATCC 3′ reverse primers directed to the variable regions V3–V4 of the 16S rRNA gene. All Illumina reads were analyzed using the FastaQC software in order to assess sequence quality. Further data processing including trimming and 16S rRNA analysis and visualization was performed with a workflow based on the mothur software package (1.39.5 version). Briefly, chimeras were detected using the software UCHIME version 4.2 (https://drive5.com/uchime, effective tags obtained) and sequences were aligned and clustered into operational taxonomic units (OTUs) with an identity cut of 80%. The total count threshold was set at 0.005% using the Greengenes database (13).

#### Microbial Functional Analysis: PICRUSt

Within the metagenomic study, the analysis of the most represented functions of the microbial community was conducted. For this purpose, PICRUSt (version 1.1.3) was used for comparing the predicted functional profiles from the anterior and posterior intestines of gilthead seabream fed both administered diets. PICRUSt is a bioinformatics software designed to predict the functional profile of a microbial community based on the study of the 16S rRNA. Reads from 12 samples (three samples from intestine sample and treatment) were filtered by rarefaction curves from 46,331 reads, and singletons were removed. A total of 45,844 sequences, which clustered into 103 OTUs identified in the Greengenes database, were used for additional bioinformatics analysis (Supplementary Figure 1). Sequencing data were introduced into the PICRUSt bioinformatics software, and samples were normalized to the number of copies of the 16S rRNA. Functional metagenomes for each sample were predicted from the Kyoto Encyclopedia of Genes and Genomes (KEGG) catalog and collapsed to specified KEGG levels.

### Statistics

Differences between biometrical parameters of both experimental dietary groups were analyzed through an unpaired *t*-test assuming data homoscedasticity (GraphPad PRISM 7.00; *p* < 0.05).

Raw data extracted from microarrays were imported and analyzed using GeneSpring version 14.5 GX software (Agilent Technologies). The 75th percentile normalization was used to standardize the arrays for comparisons, and data were filtered by expression. The DEGs were obtained from a gene-level differential expression analysis. An unpaired *t*-test was conducted without correction (*p* < 0.05) to identify DEGs between dietary treatments. The DEGs were grouped according to their fold-change value (FC, *p* < 0.05) and represented using the GraphPad PRISM software. The Principal Component Analysis (PCA) on conditions was carried out using GeneSpring software; four eigenvectors were calculated using a covariance matrix to describe the aggrupation of the control and EOs groups in a 3D plot. The gene expression values (log2-expression ratios) were represented by a hierarchical clustering heatmap analysis (MeV software v4.0), with Pearson distance and average linkage (9).

All data analysis of the intestinal microbiota was processed using Phyloseq and Vegan libraries in R statistical package. Readings were normalized based on rarefaction curves (46,331 reads) and singletons were removed. In addition, it was calculated the coverage using the Good's coverage coefficient, as well as the ecological indexes. Alpha diversity was estimated using the Chao1, Shannon, and Simpson indices, to assess taxonomic wealth, diversity, and dominance, respectively. For statistical analyses between diversity indices, the *t*-test (*p* < 0.05) was used; while the taxonomic comparison was carried out using the R package DESeq2 (*p* < 0.01). Differences in the functional prediction between diets were made using the ANOVA multiple comparison test with the Tukey-Kramer correction (corrected *p* < 0.05).

## Results

### Growth Performance

At the end of the study, no significant differences were observed between fish fed the EOs-supplemented diet and the control diet in terms of somatic growth (BWf = 150.8 ± 14.9 vs. 157.8 ± 14.2 g; SLf = 17.1 ± 0.6 v*s*. 17.3 ± 0.6 mm), daily growth rates in terms of BW (SGR_BW_ = 2.03 ± 0.01 vs. 2.12 ± 0.07% BW/day) and Fulton's condition factor (K = 3.0 ± 0.1 vs. 3.1 ± 0.1), respectively (Unpaired *t-*test, *p* > 0.05). A survival rate of 96 and 92% was recorded for the EOs-supplemented and control diets, respectively.

### Microarrays and Gut Transcripteractome

**Figure 1:**
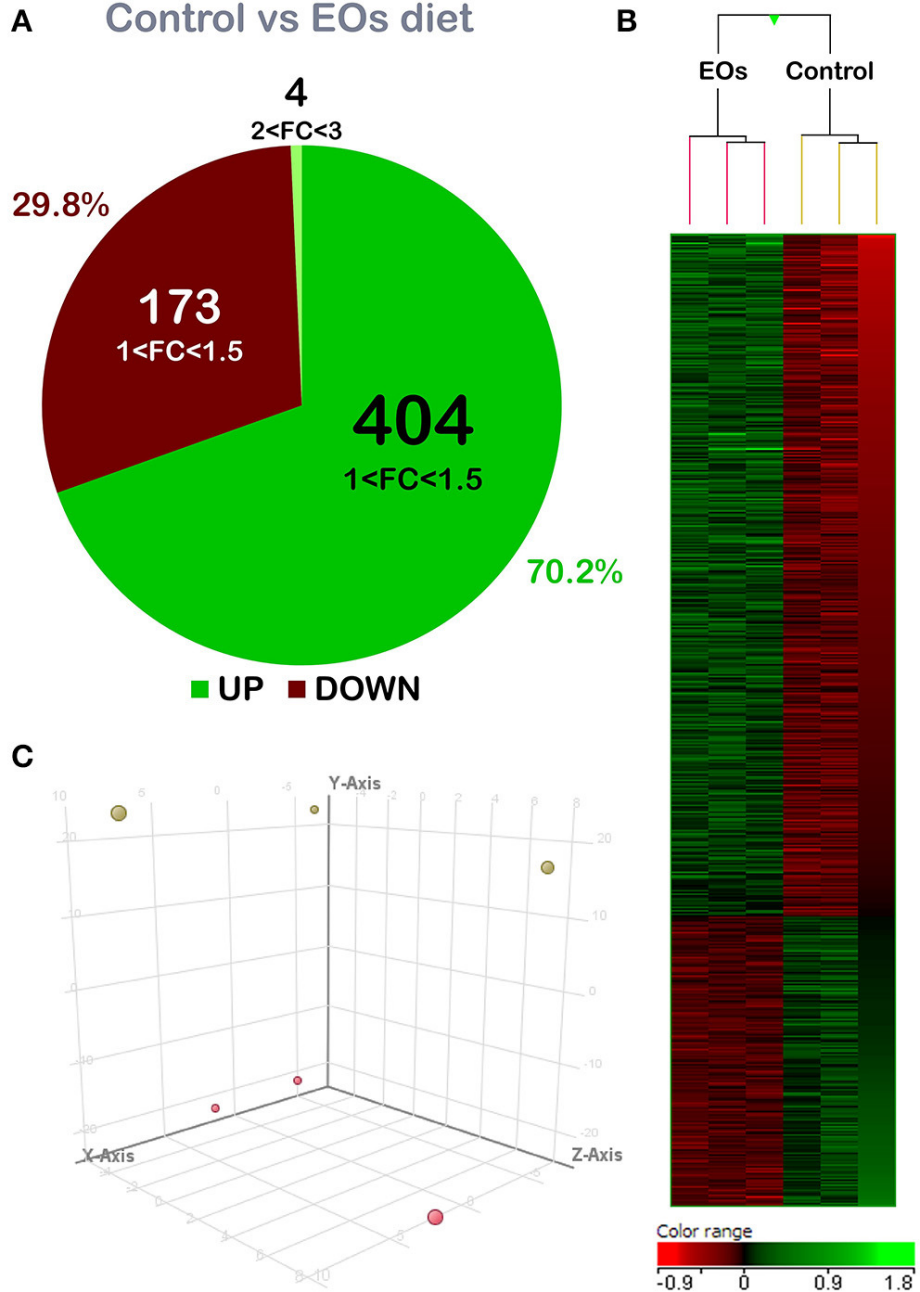
Differential expression analysis of the gilthead seabream (*Sparus aurata*) mid-anterior intestine transcriptomic response to the garlic, carvacrol, and thymol essential oils (EOs)-supplemented diet. **(A)** Comparing both groups, 581 differentially expressed genes (DEGs) (*p* < 0.05) were found. From these, 408 genes were upregulated: 404 mainly concentrated in the 1.0–1.5-fold change (FC) interval; and 4 DEGs were grouped in the 2.0 ≤ FC ≤ 3.0 interval. Additionally, 173 genes were downregulated (*p* < 0.05) and grouped in the range −1.5 ≤ FC ≤ −1.0. **(B)** Hierarchical clustering of the gilthead seabream mid-anterior intestine transcriptomic response for the control diet and EOs-supplemented diet, based on similitude patterns of the DEGs detected from three sample pools per dietary group. Data of the six microarrays are depicted, one for each represented pool. Both increased and decreased gene expression patterns are shown in green and red, respectively. All transcripts represented are statistically significant (*p* < 0.05). **(C)** Principal component analysis (PCA) of the DEGs of gilthead seabream intestine response to the control diet (yellow) and EOs-supplemented diet (red). PC1, PC2, and PC3 components loadings (Y-axis = component 1, 79.03% of variation; X-axis = component 2, 6.26% of variation; Z-axis = component 3, 6.03% variation). For DEGs details, please see also Supplementary Table 1.

A total of 581 DEGs were found when comparing the transcriptomic profiling of the intestine from fish fed both diets (*p* < 0.05; Supplementary Table 1). The detailed analysis of gene FC revealed that genes were mostly upregulated in the fish fed the diet containing the functional additive (70.2% of DEGs), although its modulation was moderate in terms of FC intensity. In particular, 408 of the abovementioned DEGs were upregulated, with 404 of them within the 1.0 < FC < 1.5 interval. The remaining four DEGs were grouped within the 2.0 ≤ FC ≤ 3.0 interval. In addition, 173 DEGs were downregulated (29.8% of DEGs; *p* < 0.05) and all of them were grouped within the −1.5 ≤ FC ≤ −1.0 interval (Figure 1A). Common segregation among the pool samples within the same dietary treatment was observed in the hierarchical clustering of the intestine transcriptomic response based on correlation patterns from the DEGs response (*p* < 0.05) (Figure 1B). PCA analysis confirmed the differential transcriptomic profile among dietary treatments. The first component (Y-axis) accounted for 79.03% of variation; the second and third components (X-Axis and Z-axis, respectively) accounted for 6.26 and 6.03% of the variation; these three components show the perfect separation between the control diet and the fish fed with the additive (Figure 1C).

**Figure 2:**
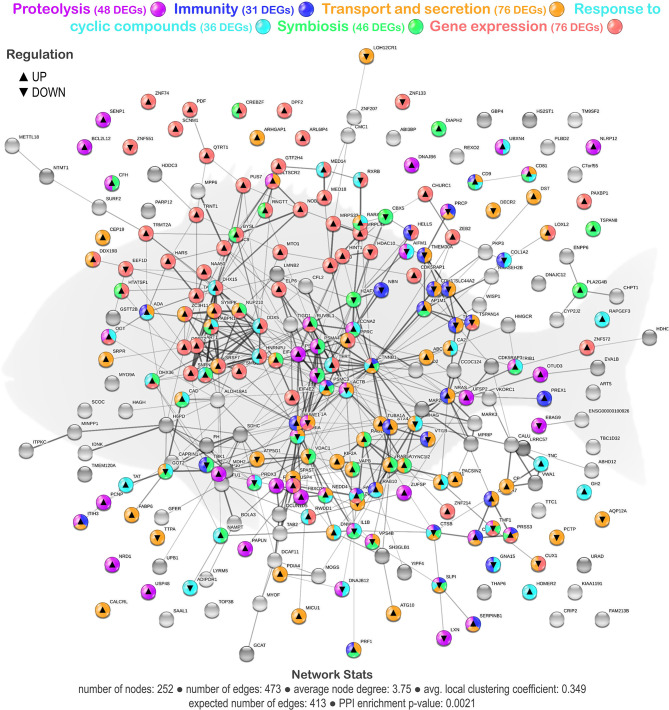
Transcripteractome of the differentially expressed genes (DEGs) in the mid-anterior intestine of juvenile gilthead seabream (*S. aurata*) fed the garlic, carvacrol, and thymol essential oils (EOs)-supplemented diet (see also Supplementary Tables and Supplementary Figures 2–8). Protein–Protein Interactions Network (PPI) network nodes colors indicate the six representative processes identified from the functional enrichment analysis for each DEG represented—proteolysis, immunity, transport, and secretion, response to cyclic compounds, symbiosis, and gene expression. ▴ nodes represent upregulated genes and ▾ nodes represent downregulated genes. Graphic keys and network stats are indicated in the graphical figure legend.

From the whole set of DEGs, a functional network analysis was performed. The transcripteractome showed 252 coding proteins (nodes) with 473 interactions (edges). The remaining 329 DEGs (annotated as unknown genes) were excluded from the analysis. Based on the 70 GO terms obtained from the enrichment analysis (Supplementary Table 2), 6 main representative groups for the biological processes were identified in the transcripteractome: (1) proteolysis; (2) immunity; (3) transport and secretion; (4) response to cyclic compounds; (5) symbiosis; and (6) gene expression (Figure 2).

The tested functional feed additive resulted in the positive regulation of biological processes related to the proteolysis category (32 upregulated genes and 16 downregulated genes; Supplementary Figure 2; Supplementary Table 3). Several biological processes were considered, namely, “proteolysis,” “regulation of proteolysis,” “regulation of peptidase activity,” “regulation of endopeptidase activity,” and “protein deubiquitination.” In addition, biological processes associated with immunity showed a more balanced regulation in fish fed the EOs-supplemented diet (17 upregulated genes and 14 downregulated genes; Supplementary Figure 3; Supplementary Table 4). Several relevant GOs related to immunity were obtained, such as “cell activation,” “leukocyte activation,” “leukocyte activation involved in immune response,” “neutrophil activation,” “neutrophil degranulation,” and “regulated exocytosis.” The tested functional feed additive also favored biological processes associated with transport and secretion (50 upregulated genes and 26 downregulated genes; Supplementary Figure 4; Supplementary Table 5). Among them, “transport,” “intracellular transport,” “vesicle-mediated transport,” and “secretion” processes were identified in the functional network. Moreover, some biological processes in the category of the response to cyclic compounds were positively affected by the dietary inclusion of EOs (25 upregulated genes and 11 downregulated genes; Supplementary Figures 5, 6; Supplementary Table 6). In particular, the following processes were evidenced, such as the “response to organic cyclic compound,” “cellular response to organic cyclic compound,” “response to lipid,” “cellular response to lipid,” “response to hormone,” “response to steroid hormone,” “cellular response to hormone stimulus,” “cellular response to steroid hormone stimulus,” “response to alkaloid,” “response to nitrogen compound,” and “response to organonitrogen compound.” Furthermore, symbiosis correlated biological processes, such as “symbiont process,” “interspecies interaction between organisms,” and “multi-organism process,” were positively modulated (33 upregulated genes and 13 downregulated genes) in the intestine of fish fed the diet containing the functional feed additive (Supplementary Figure 7; Supplementary Table 7). Finally, the biological processes associated with gene expression and RNA processing (62 upregulated genes and 10 downregulated genes; Supplementary Figure 8; Supplementary Table 8), among them “gene expression,” “RNA processing,” “RNA splicing,” “messenger RNA (mRNA) processing,” “mRNA metabolic process,” “mRNA export from nucleus,” and “ribonucleoprotein complex export from nucleus” were observed to be much more upregulated in the intestine of fish fed the EOs-supplemented diet than in the control group.

A reasonable number of genes were observed to be shared among the immunity category and the other categories of gene expression/RNA processing (16%), proteolysis (29%), transport/secretion (77%), response to cyclic compounds (32%), and symbiosis (32%), suggesting a strong relationship between these biological processes that favors the host mucosal tissue immunity in response to the dietary EOs. Additionally, from the total DEGs obtained from the transcriptomic profile of the fish fed the EOs-supplemented diet, a set of genes was selected based on their expression and biological relevance (Table 2) in order to assess the participation of the intestine transcriptional regulation upon the observed microbiota modulation.

**Table 2:** List of selected differentially expressed genes (DEGs) of the mid-anterior intestine of juvenile gilthead seabream (*Sparus aurata*) fed a diet supplemented with a blend of garlic, carvacrol, and thymol essential oils (EOs).

**Gene name**	**Acronym**	**FC**	***P*-value**
**PROTEOLYSIS AND DEUBIQUITINATION**			
UFM1 Specific Peptidase 2	*ufsp2*	1.621	0.012
Proteasome 20S Subunit Alpha 6	*psma6*	1.491	0.014
Ubiquitin Specific Peptidase 10	*usp10*	1.490	0.026
Proteasome 26S Subunit, ATPase 3	*psmc3*	1.413	0.040
Ubiquitin Specific Peptidase 48	*usp48*	1.359	0.036
Proteasome 20S Subunit Alpha 4	*psma4*	1.289	0.027
OTU Deubiquitinase 3	*otud3*	1.256	0.035
Ubiquitin Specific Peptidase 4	*usp4*	1.103	0.007
Interleukin 1 Beta	*il-1β*	−1.167	0.025
NFKB Inhibitor Alpha	*nfkbia*	−1.327	0.047
Proteasome 20S Subunit Beta 6	*psmb6*	−1.495	0.048
**IMMUNITY**			
Leukocyte Elastase Inhibitor	*serpinb1*	1.561	0.024
Adenosine Deaminase	*ada*	1.517	0.025
Phosphatidylinositol-3,4,5-Trisphosphate Dependent Rac Exchange Factor 1	*prex1*	1.478	0.018
CD9 Molecule	*cd9*	1.283	0.033
CD81 Molecule	*cd81*	1.279	0.018
Perforin 1	*prf1*	1.181	0.034
Cathepsin B	*ctsb*	−1.340	0.031
**TRANSPORT AND SECRETION**			
Fatty Acid Binding Protein 6	*fabp6*	2.659	0.044
Serine Protease 3	*prss3*	1.824	0.002
RAB5A, Member RAS Oncogene Family	*rab5a*	1.668	0.023
RAB10, Member RAS Oncogene Family	*rab10*	1.456	0.030
Rho GTPase Activating Protein 1	*arhgap1*	1.363	0.048
NRAS Proto-Oncogene, GTPase	*nras*	1.257	0.010
RAB1A, Member RAS Oncogene Family	*rab1a*	1.238	0.025
Hypoxia Inducible Factor 1 Subunit Alpha	*hif1a*	−1.259	0.002
**RESPONSE TO CYCLIC COMPOUNDS**			
Carbonic Anhydrase 2	*ca2*	2.089	0.046
Tribbles Pseudokinase 1	*trib1*	1.740	0.011
Glutathione S-Transferase Theta 2B	*gstt2b*	1.456	0.018
Cytochrome P450 Family 2 Subfamily J Member 2	*cyp2j2*	1.284	0.045
Growth Hormone 2	*gh2*	1.283	0.032
ATPase Na+/K+ Transporting Subunit Alpha 1	*atp1a1*	1.271	0.022
Peroxiredoxin 3	*prdx3*	−1.220	0.009
Glutamic-Oxaloacetic Transaminase 2	*got2*	−1.254	0.008
Adiponectin Receptor 1	*adipor1*	−1.262	0.041
Cathepsin B	*ctsb*	−1.340	0.031
**SYMBIOSIS**			
Retinoic Acid Receptor Alpha	*rara*	1.164	0.003
Retinoid X Receptor Beta	*rxrb*	−1.172	0.030
**GENE EXPRESSION**			
Zinc Finger Protein 572	*znf572*	1.389	0.019
CDK5 Regulatory Subunit Associated Protein 3	*cdk5rap3*	1.341	0.014
Zinc Finger E-Box Binding Homeobox 2	*zeb2*	1.315	0.003
Heterogeneous Nuclear Ribonucleoprotein U	*hnrnpu*	1.284	0.012
Cellular Communication Network Factor 4	*wisp1*	1.280	0.040
NOP53 Ribosome Biogenesis Factor	*gltscr2*	1.263	0.034
Zinc Finger Protein 74	*znf74*	1.256	0.038
Pre-MRNA Processing Factor 8	*prpf8*	1.214	0.005
Zinc Finger CCCH-Type Containing 11A	*zc3h11a*	1.207	0.023
Zinc Finger Protein 214	*znf214*	1.201	0.008
Small Nuclear Ribonucleoprotein U5 Subunit 200	*snrnp200*	1.192	0.008
F-Box Protein 31	*fbxo31*	1.178	0.021
Spliceosome Associated Factor 1, Recruiter Of U4/U6.U5 Tri-SnRNP	*sart1*	1.155	0.005
Zinc Finger Protein 133	*znf133*	−1.127	0.006
Zinc Finger Protein 551	*znf551*	−1.168	0.001
Nibrin	*nbn*	−1.238	0.031

*Genes were arranged according to six representative processes identified from the functional enrichment analysis. Gene description, respective acronym, fold-change intensity (FC), modulation (green: upregulation; red: downregulation), and p-value are described*.

### Intestine Microbiota Analysis

No variation was registered on the alpha diversity indices of the intestinal microbiota, regardless of the region of the intestine considered (Table 3; *p* > 0.05). However, the coefficient of variation (CV) for the Chao1 index was higher in the anterior (CV = 18.7 vs. 5.2%) and posterior (CV = 23.9 vs. 12.3%) intestinal segments of fish fed the diet containing the functional feed additive (Supplementary Figure 9). Similarly, the Shannon and Simpson diversity indexes were neither affected by the inclusion of the functional feed additive in the diet. Library coverage was calculated using Good's coverage index with a result of 99.98 ± 0.01.

**Table 3:** Alpha diversity of bacterial communities in the anterior and posterior intestinal tract sections of gilthead seabream (*Sparus aurata*) fed a control and the diet supplemented with a blend of garlic, carvacrol, and thymol essential oils (EOs).

			**Chao1**	**Shannon**	**Simpson**
Intestine	Anterior	Control	64.583 ± 3.357	2.513 ± 0.231	0.863 ± 0.046
		EOs diet	69.437 ± 13.013	2.288 ± 0.470	0.838 ± 0.093
	Posterior	Control	58.167 ± 7.182	2.401 ± 0.550	0.807 ± 0.164
		EOs diet	62.770 ± 14.983	2.363 ± 0.220	0.863 ± 0.039

**Figure 3:**
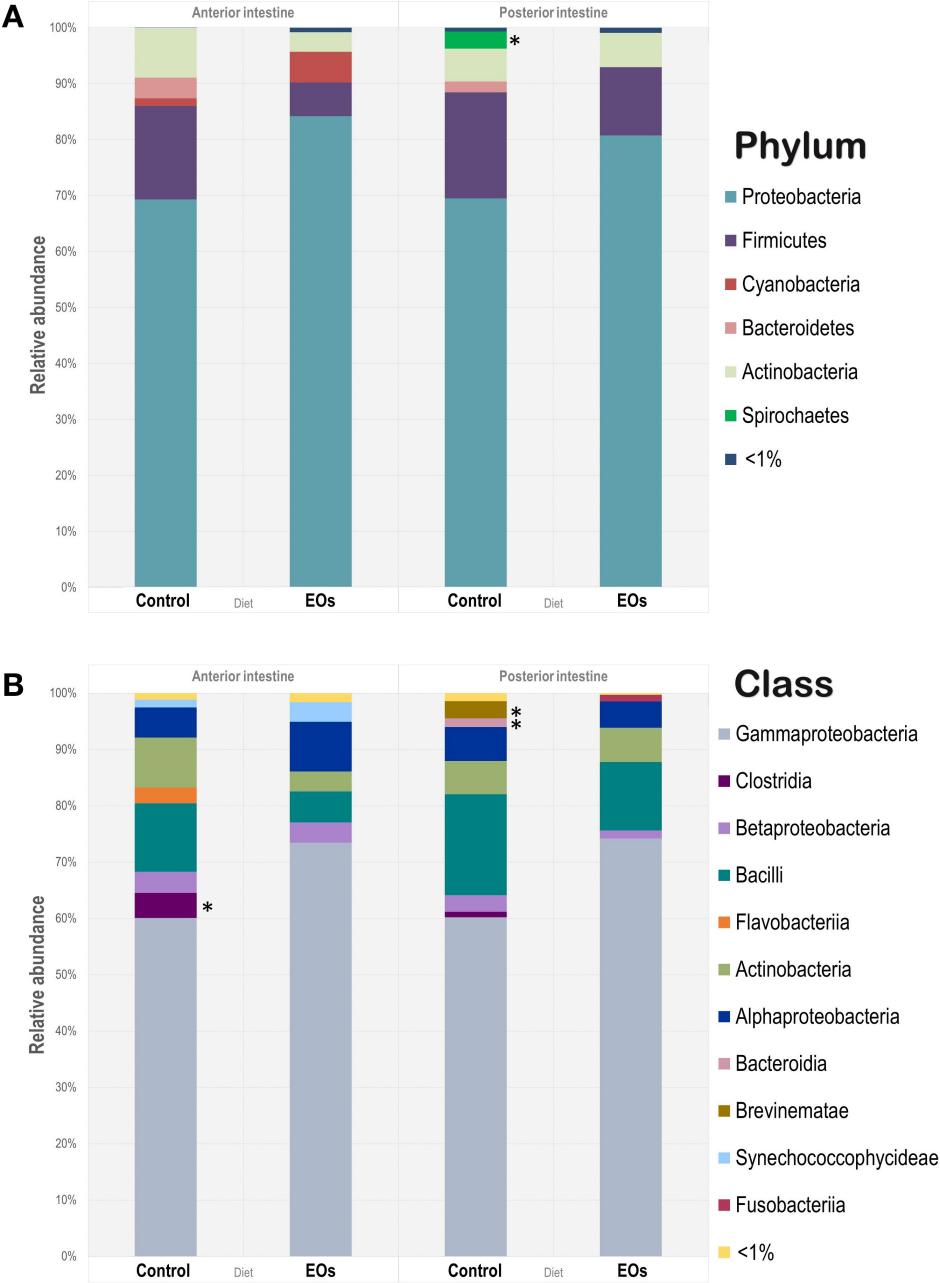
Microbiota composition at phylum **(A)** and class **(B)** level of the anterior and posterior sections of the intestinal tract of gilthead seabream (*S. aurata*) fed control and garlic, carvacrol, and thymol essential oils (EOs)-supplemented diet. Asterisk (*) indicate significant differences among diets (*p* > 0.05).

The relative abundance of microbial taxa at the phylum level is shown in Figure 3A. Proteobacteria, Firmicutes, and Actinobacteria were commonly found in all samples regardless of the dietary condition and region of the intestine considered. However, only the phylum Spirochaetes showed significantly lower abundances in the posterior intestine in fish fed the diet supplemented with the functional feed additive (*p* < 0.05). At class level, γ*-Protebacteria* were the dominant group (60–73%; *p* < 0.05) in all samples assayed, whereas the abundance of *Clostridia* in the anterior intestine, and *Brevinematae* and *Bacteroidia* in the posterior intestine significantly decreased in fish fed the diet containing the blend of EOs (*t*-test, *p* < 0.05; Figure 3B).

**Figure 4:**
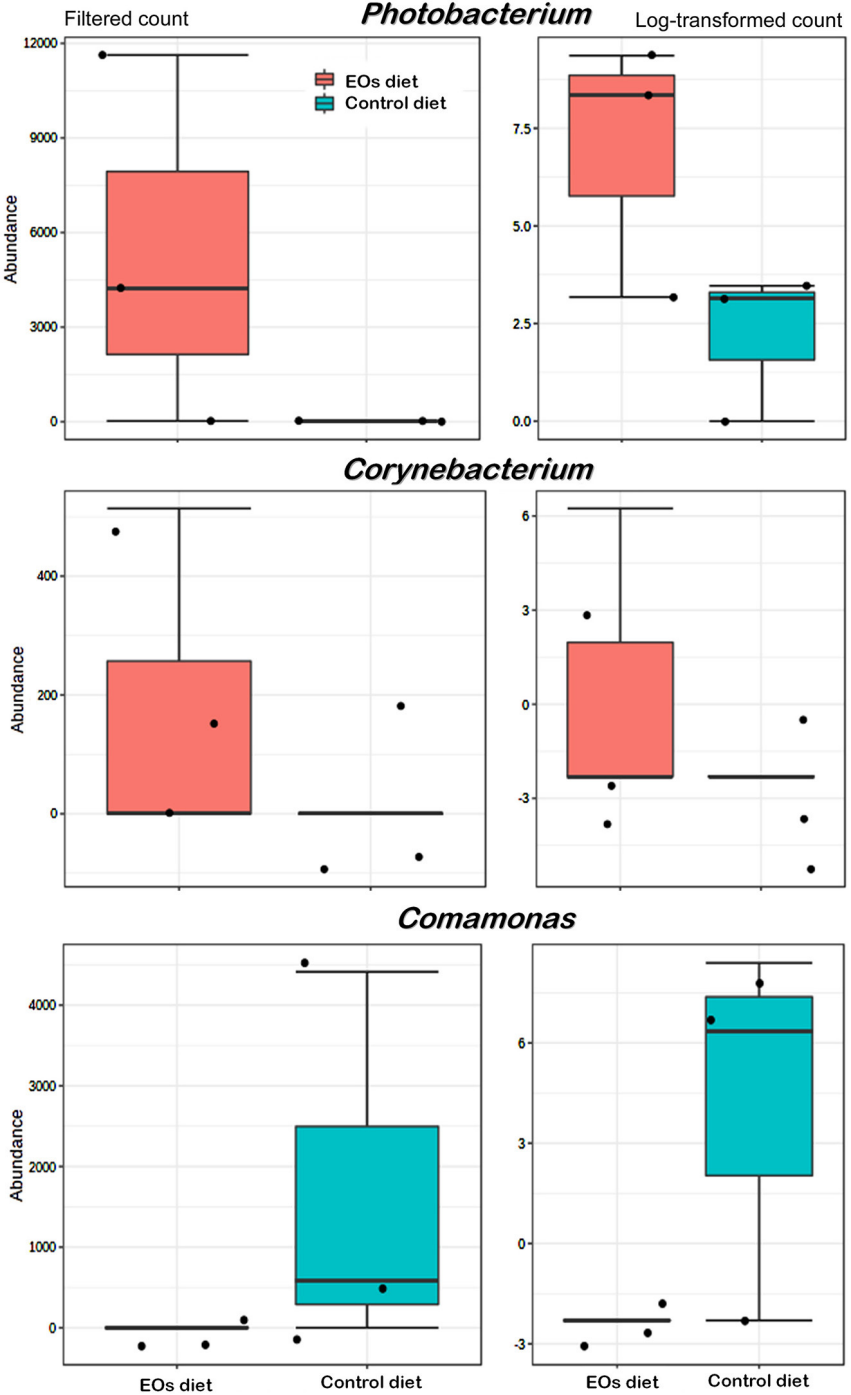
Average abundances of genera showing significant differences (*p* < 0.01) in the anterior intestine of gilthead seabream (*S. aurata*) fed the garlic, carvacrol, and thymol essential oils (EOs)-supplemented diet in comparison with fish receiving the control diet.

**Figure 5:**
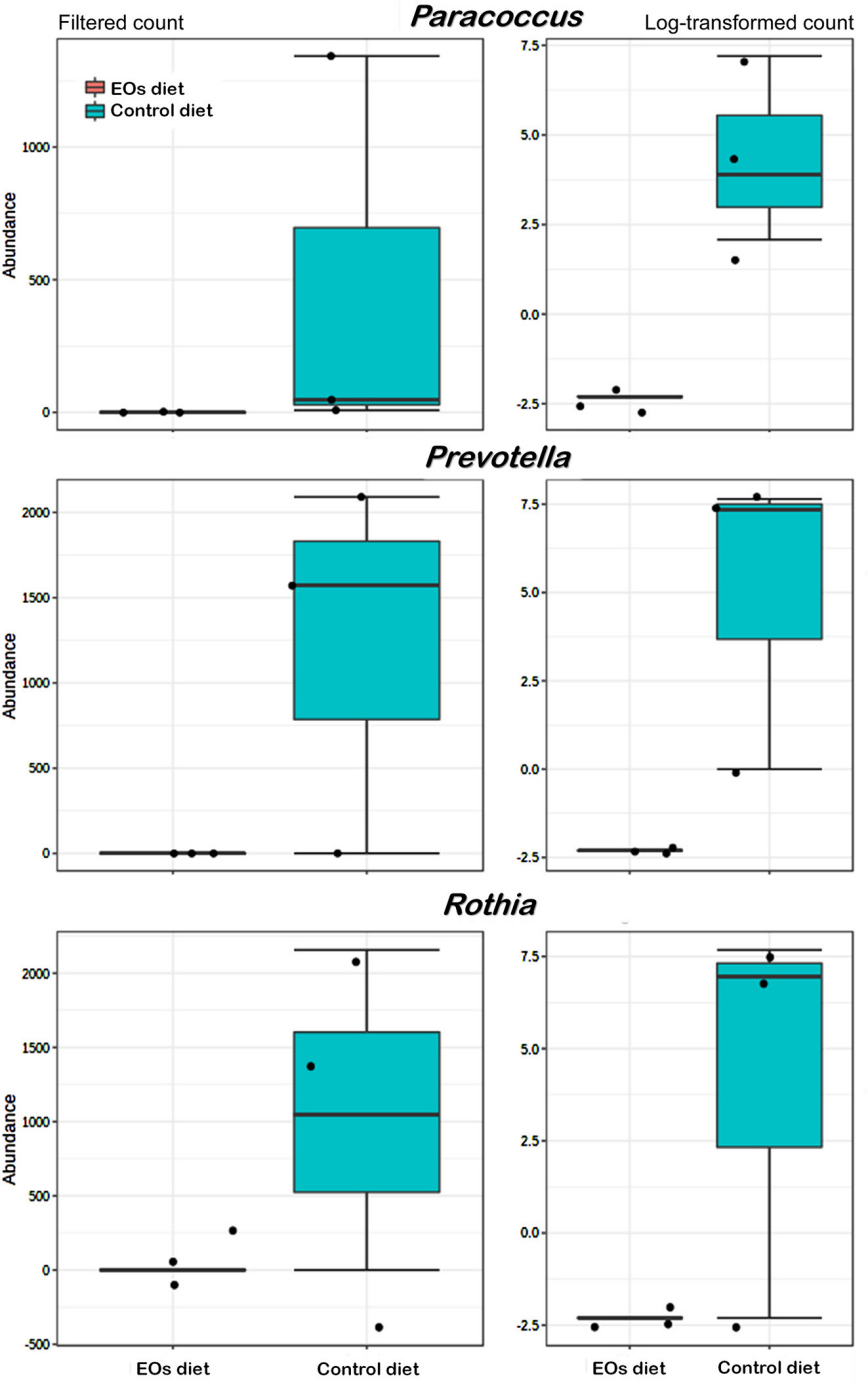
Average abundance of genera showing significant differences (*p* < 0.01) in the posterior intestine of gilthead seabream (*S. aurata*) fed the garlic, carvacrol, and thymol essential oils (EOs)-supplemented diet in comparison with fish receiving the control diet.

At the genus taxonomic level, bacterial abundance (relative abundance > 1%) was significantly affected by the functional feed additive tested (*t*-test, *p* < 0.05). In particular, the anterior intestine of gilthead seabream fed the EOs-supplemented diet showed a significant increase (*t*-test, *p* < 0.05) in *Photobacterium* (γ-Proteobacteria, *Vibronaceae*) and *Corynebacterium* (Actinobacteria, *Corynebacteriaceae*) abundance whereas a reduction in *Comamonas* (Proteobacteria, *Comamonadaceae*) was also found (*t*-test, *p* < 0.05; Figure 4). Regarding the posterior intestine, a significant decrease in the abundance of the genera *Paracoccus* (Proteobacteria, *Rhodobacteraceae*), *Prevotella* (Bacteroidetes, *Bacteroidaceae*), and *Rothia* (Actinobacteria, *Micrococcaceae*) was also detected in fish fed the functional feed additive (*t*-test, *p* < 0.05; Figure 5).

**Figure 6:**
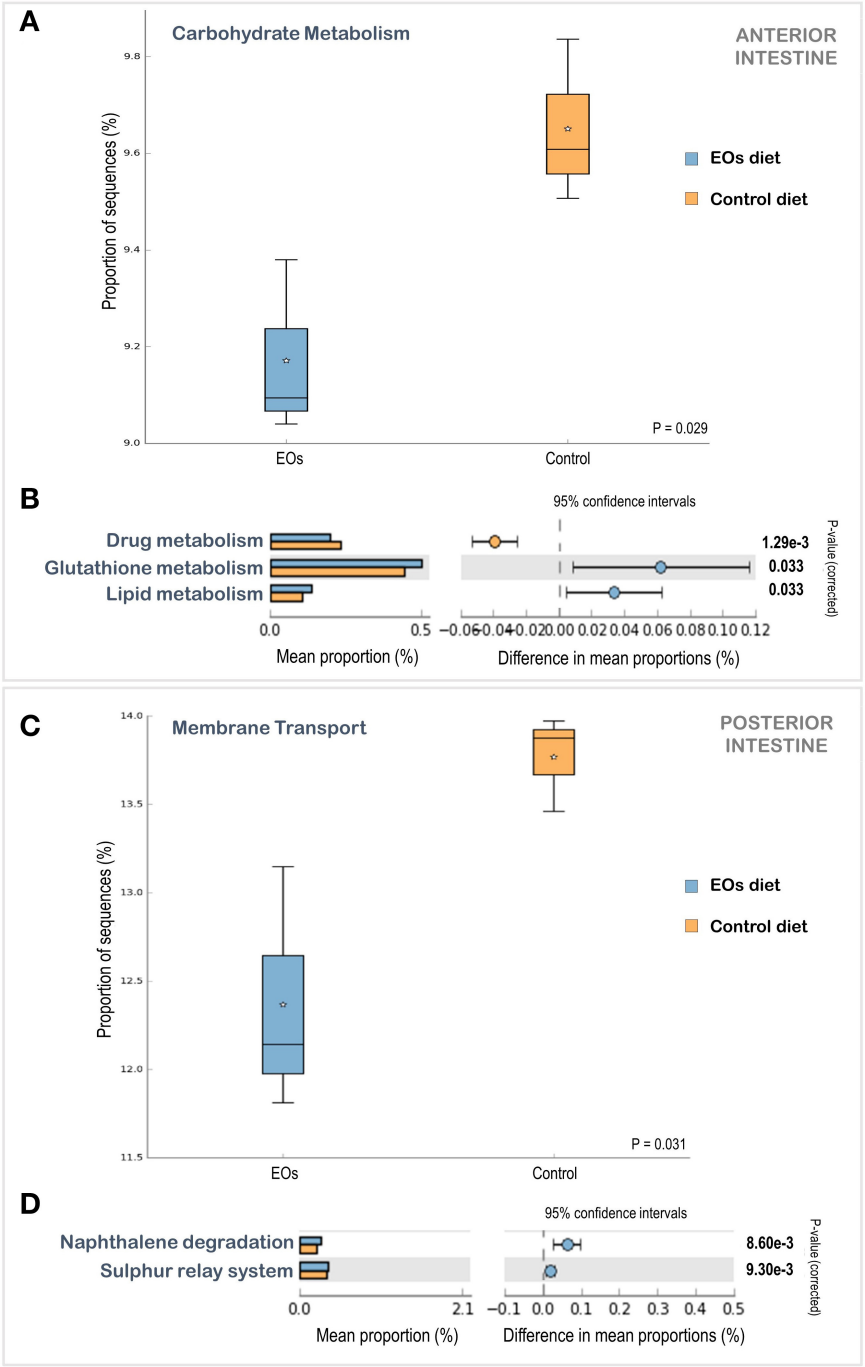
Comparison of the relative abundance of PICRUSt generated functions profile in the anterior **(A,B)** and posterior **(C,D)** intestinal microbiota of gilthead seabream (*S. aurata*) fed the control and the garlic, carvacrol, and thymol essential oils (EOs)-supplemented diet. Box plots show the relative abundances of significant changes in level 2 Kyoto Encyclopedia of Genes and Genomes (KEGG) pathways in the anterior **(A)** and posterior **(C)** intestinal microbiota. Box plot central lines indicate the median and star symbols indicate the mean of the data. Significant KEGG pathways at level 3 pathways in the anterior **(B)** and posterior **(D)** intestinal microbiota, by ANOVA with a *post-hoc* Tukey–Kramer multiple-comparison test (*p* < 0.05).

The PICRUSt analysis enabled the prediction of the functional capacities of the microbial communities detected in the gilthead seabream intestine based on the treatment applied. The low Nearest Sequenced Taxon Index (NSTI) value (0.04 ± 0.02) from the PICRUSt analysis indicated a good prediction accuracy. The functional analysis of KEGG pathways revealed significant differences at second- and third-level classification KEGG pathways in both sections of the intestine (ANOVA, *p* < 0.05). Regarding the anterior intestine and considering the second-level classification of KEGG pathways, a reduction in carbohydrate metabolism in fish fed the diet supplemented with the functional feed additive was obtained (ANOVA, *p* < 0.05) (Figure 6A). When considering the third-level classification of KEGG pathways in the anterior intestine, a larger proportion of sequences associated with glutathione and lipid metabolism, and a reduction of sequences related to drug metabolism was found in fish fed the EOs-supplemented diet (Figure 6B). Regarding the posterior intestine, processes related to membrane transport at the second-level classification of KEGG pathways were significantly reduced in fish fed the EOs-supplemented diet (Figure 6C), while processes related to the sulfur relay system and naphthalene degradation at the third-level classification of KEGG pathways were significantly enhanced in fish fed the EOs-supplemented diet (Figure 6D).

## Discussion

The fish intestine is a complex multifunctional organ. In addition to diet digestion and nutrient absorption functions, this organ is critical for other key physiological mechanisms including water and electrolyte balance, endocrine regulation upon digestion and metabolism, and immunity (7), as well as for the establishment of commensal microbiota (2, 14). As one of the main portals of pathogens' entry into the organism, and due to its intricate immune system associated to a lymphoid tissue that allows microbial colonization, the intestine of farmed fish is a target tissue for dietary manipulations (2, 7). We aimed to describe the effect of the dietary combination of garlic, carvacrol, and thymol EOs in the intestinal mucosa of gilthead seabream at a transcriptional level and its impact on gut microbiota modulation.

### Effect of Garlic, Carvacrol, and Thymol EOs Additive on Proteolysis, Protein Deubiquitination, and Inflammatory Regulation

The dietary supplementation of EOs affected the regulation of several genes associated with proteolytic pathways, peptidases activity, and protein deubiquitination. In particular, several genes coding for proteasomes (*psma6, psmc3, psma4*, and *psmb6*) and ubiquitin peptidases (*usp4, usp10, usp48, otud3*, and *ufsp2*) were activated by the dietary supplementation of EOs. Protein ubiquitination and subsequent proteolysis and degradation by the proteasome are important mechanisms in the regulation of the cell cycle, cell growth and differentiation, gene transcription, signal transduction, and apoptosis, including tissue regeneration (15). As important factors for the maintenance of intestinal epithelial integrity and immune homeostasis, the ubiquitin-proteasome proteolytic system plays a pivotal role in the activation of the nuclear factor kB (NF-kB) pathway. The NF-kB pathway is involved in the transcriptional regulation of several proinflammatory genes, playing a critical role in regulating the survival, activation, and differentiation of innate and adaptive immune cells (16). As an important feedback regulatory mechanism following NF-kB activation, the NFKB inhibitor alpha (*nfkbia*), also known as *IkB*α, is one of the first genes to be activated (16). In our transcriptional analysis, *nfkbia* was downregulated. In regard to the NF-kB signaling pathway, we also registered the upregulation of several deubiquitination-related genes, such as the referred ubiquitin peptidases, that could be blocking the ubiquitination and degradation of the NF-kB inhibitors. This mechanism might be accompanied by commensal γ-*Protebacteria* strains, the dominant class in our study, that prevent or limit epithelial gut inflammation (17); thus, evidencing the close cooperation between gut and microbiota in the inflammatory regulation in response to dietary shifts. Accordingly, the proinflammatory cytokine interleukin-1 beta (*il-1*β) was downregulated in the fish fed the EOs-supplemented diet, corroborating the reduction of the transcription of proinflammatory genes mediated by the NF-kB pathway. Collectively, these data suggest a direct and selective regulation of the NF-kB and ubiquitin-proteasome pathways established through the interaction between the transcriptome response and their commensal bacteria in the gut of sea bream fed the EOs-supplemented diet.

### Effect of Garlic, Carvacrol, and Thymol EOs Additive on Immune Effector Processes

The dietary administration of EOs showed the modulation of several biological processes related to innate immune effector cells, such as “leukocyte activation,” “leukocyte activation involved in immune response,” “neutrophil activation,” and “neutrophil degranulation.” In the case of neutrophils, their main function is the control of microorganisms that cross the epithelium barrier to invade the mucosa. Contrarily to mammals, fish neutrophils are not so abundantly present in the bloodstream, whereas they are stored in hematopoietic reservoirs instead, which could signify a disadvantage for rapid migration and effective resolution of infection and inflammation events (7). Thus, the results of our functional analysis regarding leukocyte activation, and granulocytes, in particular, might suggest an increased intestinal specific immune capacity promoted by the tested functional diet. In fact, the dietary supplementation of garlic or its bioactive compounds (18), carvacrol, and/or thymol (19) have been reported to increase the number of white blood cells and other immune parameters in several cultivated fish species. Similarly to our results, the activation of the degranulation transcriptional response of the neutrophils was previously observed in the gills of gilthead seabream fed the same EOs-supplemented diet and attributed to its increased defense capacity against a monogenean helminth parasite infection (9).

Further analyses on immune-related processes modulated by the tested EOs also showed an increase of the expression in *prex1*, a gene coding for the phosphatidylinositol 3,4,5-trisphosphate-dependent Rac exchanger 1 that regulates adhesion, migration, tissue recruitment, and reactive oxygen species (ROS) formation in the neutrophils (20). In addition, the leukocyte elastase inhibitor gene (*serpinb1*) was also upregulated in the intestine of fish fed the EOs-supplemented diet. This gene encodes a serine protease inhibitor that specifically inhibits neutrophil elastase, cathepsin G, and proteinase-3 present in the neutrophil granules; thus, protecting not only tissues from damage at inflammatory sites during stress or infection, but also the neutrophil itself (21). SERPINB1 also limits the activity of inflammatory caspases during inflammation by suppressing their caspase-recruitment domain oligomerization and enzymatic activation, representing an important regulator of tissue inflammation (22). Under current experimental conditions, these results may indicate a well-balanced intestinal immunity, where both immune effector cells activation and an anti-inflammatory response were promoted.

The expression of several genes associated with adaptive immunity was modulated by the EOs-supplemented diet as well. For instance, perforin-1 (*prf1*) was upregulated in the intestine of fish fed the EOs-diet. Perforin is a pore-forming cytolytic protein found in the granules of cytotoxic T lymphocytes (CTLs) and natural killer (NK) cells, playing a key role in killing other cells that are recognized as non-self by the immune system (23). In fish, studies have reported the upregulation of *prf1* in response to viral stimulation (24). Furthermore, the upregulation of adenosine deaminase (*ada*) was also promoted by the tested EOs-supplemented diet. In particular, *ada* acts as a positive regulator of T-cell co-activation, participates in the regulation of lymphocyte-epithelial cell adhesion, and enhances dendritic cell immunogenicity (25, 26). Additionally, *cd9* and *cd81* were both upregulated in the fish fed the EOs-supplemented diet. These genes encode tetraspanins, key players in the processes of adhesion, extravasation, and recruitment of leukocytes into inflammation sites, regulating several steps of the immune response (27). CD9 and CD81 were found to be extensively present in Atlantic salmon (*Salmo salar*) IgM^+^ B-cells (28). Last but not least, tetraspanins were considered to be required for bacteria adhesion to the epithelial cells (29); which may be in agreement with the presence of both DEGs in the symbiosis-related processes from our transcriptional analysis, as discussed below. Therefore, the regulation of genes involved in both B and T lymphocytes activity may suggest the stimulation of not only the innate, but also the adaptive immune response as well, although further research is needed to confirm this hypothesis.

The proportion of up to downregulated genes related to an immune response was not as marked as the observed for the remaining biological processes activated by the inclusion of EOs in diet, indicating an effective and balanced proinflammatory and anti-inflammatory regulation of the induced immune response, as previously suggested. Nonetheless, it is legitimate to assume that due to its immunostimulatory characteristics, the EOs-based functional feed might have an impact on the composition of the gilthead seabream intestinal microbiota, which in turn may also have played a critical role in mediating the abovementioned immune response. In fact, numerous studies have indicated that diet is an important factor in the modulation of the gut microbiome composition in vertebrates, dictating also the role of that microbiome in fish health status (2). Regarding the EOs tested, garlic (4), carvacrol, and/or thymol (5) were previously reported to modulate fish microbiota composition, exerting beneficial effects (30), and improving significantly its resistance to *Vibrio anguillarum* after intestinal infection and stress challenge (31). The administration of similar functional diets with immunostimulatory and/or antimicrobial properties have been also reported to reduce gut microbial diversity (30); however, in our study, alpha diversity was not significantly altered by the dietary EOs, which may be associated with the heterogeneity of analyzed samples, as observed in CV values for the Chao1 index. Under present experimental conditions, the only phylum that showed significant differences among dietary treatments was the Spirochaetes. This phylum contains important gut pathogenic species, such as *Brachyspira* species, for livestock and humans (32). Nonetheless, the impact of this phylum modulation upon gilthead seabream intestine homeostasis is not clear yet, and further studies should be performed in order to assess which members of the phylum could be participating in the immune response observed.

### Effect of Garlic, Carvacrol, and Thymol EOs Additive on Immune-Related Transport and Secretion Processes

The obtained immune-related biological processes were observed to share 77% of DEGs with the transport and secretion category, which genes were observed to be positively affected, in its majority, by the dietary EOs. The substantial amount of DEGs shared among the two categories clearly indicates a common role in the overall observed transcriptional response. In this sense, similar vesicle-mediated transport processes associated with active biogenesis and neutrophil-mediated immune response were observed in the gills of gilthead seabream fed the same EOs-supplemented diet (9), which seemed to indicate a similar action of this functional additive on different mucosal tissues. Epithelial cells are also directly involved in the initiation of the immune response, such as the one mediated by neutrophils. Accordingly, several genes encoding RAS-related GTPases (*nras, rab1a, rab5a, rab10*, and *arhgap1*), recognized as leading regulators of membrane trafficking directing immunity and inflammation cellular responses (33), were upregulated in the intestine of fish fed the functional feed additive. On the other hand, the hypoxia inducible factor 1 subunit alpha (*hif1a*) was downregulated in the fish fed the EOs-supplemented diet. HIF1a functions as a master transcriptional regulator of the adaptive response to hypoxia and it was observed to be transcriptionally induced by ROS through NF-kB (34), contributing in the intestinal mucosa to inflammatory resolution. The decrease in *hif1a* expression by the EOs dietary administration corroborated once again, that although neutrophil activation and vesicle-mediated transport processes were stimulated in the intestine of fish, inflammation derived from ROS release was probably not occurring.

Furthermore, the serine protease 3 (*prss3*) was another gene positively regulated in the intestine of fish fed the EOs-supplemented diet. This protease is involved in the synthesis of antibacterial substances (35); thus, we hypothesized that *prss3* may be involved in the regulation of intestinal immunity. PRSS3 is also a digestive protease specialized for the degradation of trypsin inhibitors (36). Trypsin inhibitors are anti-nutritional factors found in plant-protein sources that impair diet digestibility and generate digestive and metabolic disorders (37). Although the substitution of fishmeal by plant-derived protein sources was not in the scope of our study, *prss3* upregulation in the EOs-supplemented might indicate that the tested EOs could enhance diet digestibility.

Moreover, the expression of the gene coding for the fatty acid binding protein 6 (*fabp6*) was the most positively affected gene by the dietary EOs. Similarly, FABP6 was significantly increased in the intestine of gilthead seabream fed a combination of carvacrol, thymol, and a prebiotic (38), while this gene was downregulated in response to enteritis induced by a parasitic pathogen (39). Furthermore, FABP6 is involved in the transport of bile acids in ileal enterocytes (40). Besides, the influence of the intestinal microbiota on the activity of FABP6 was suggested in zebrafish, since *fabp6* expression decreased significantly after antibiotic treatment (41). Interestingly, in our study, the abundance of *Bacteroidia* class (Bacteroidetes) decreased significantly in the posterior intestine of fish fed the EOs-supplemented diet. Within the *Bacteroidia* class, *Bacteroides* genus bacterial metabolism of bile acids was observed to modulate gut T-cells homeostasis (42). Particularly, shifts toward the phylum Bacteroidetes including the *Bacteroidia* class coincides with mucosal CD4^+^ T-cell depletion and enterocyte damage (43). Therefore, the upregulation of *fabp6* and the decrease of *Bacteroidia* class might indicate a modulation of the bile acids secretion by the tested EOs, potentially affecting lipids metabolism. Since bile acids are recognized as signaling molecules between the host microbiota and the innate immunity (44), alterations in its secretion could have a role in our observed immune-related transcriptional response previously discussed. Nevertheless, further studies need to be addressed in order to evaluate the impact of the EOs-based feed additive in the digestive secretions and metabolism of gilthead seabream.

Under the transport and secretion context, gut microbiota mediate the metabolism and transport of dietary xenobiotics through the modulation of metabolites of the host or through microbial secretion (45). Accordingly, our functional analysis of KEGG pathways of the posterior intestine microbiota of individuals fed the EOs-supplemented diet showed a decrease in bacterial sequences related to membrane transport. Considering that membrane transport in prokaryotes is associated with bacterial secretion, this decrease could indicate a lower export of enzymes and bacterial toxins, commonly present in the intestinal tract of different fish species (2), representing a potential beneficial effect of the EOs administration in the gut health.

### Effect of Garlic, Carvacrol, and Thymol EOs Additive on the Response to Lipids and Hormones

Under a complex neuroendocrine regulation, the gut microbiota also regulate the metabolism of carbohydrates, lipids, and amino acids, whose composition, in turn, is susceptible to diet, health status, and drugs (46). Formerly, we suggested that the dietary administration of the EOs might affect the secretion of bile acids. If right, this could be inducing a response that could be affecting lipid metabolism and/or steroid hormone signaling. In accordance with this hypothesis, some biological processes related to responses to cyclic compounds, such as lipids and hormones, were positively affected by the inclusion of the EOs-based feed additive in the intestinal mucosa of gilthead seabream. For instance, the ATPase Na^+^/K^+^ transporting subunit alpha 1 (*atp1a1*) was significantly upregulated in the intestine of fish fed the EOs-supplemented diet. In other studies, diet-induced lipid alterations dramatically affected enterocytes lipid profile in gilthead seabream, reducing significantly Na^+^/K^+^ ATPase specific activity, suggesting a regulatory role of the lipid microenvironment on the enzyme activity (47). Additionally, tribbles pseudokinase 1 (*trib1*) gene expression was upregulated in the gilthead seabream fed the EOs-supplemented diet. This gene is known to beneficially affect plasma lipid concentration, playing also major roles in myeloid cells, improving macrophage lipid metabolism, and counteracting inflammation (48). On the other hand, the glutamic-oxaloacetic transaminase 2 (*got2*) gene was downregulated in the fish fed the EOs-supplemented diet. A study in rats suggested that leptin downregulates *got2* in adipocytes (49). Likewise, the adiponectin receptor 1 (*adipor1*) was also downregulated in the fish fed the EOs diet. Adiponectin is an essential hormone predominantly secreted by adipocytes that regulates glucose and lipid metabolism, which along with leptin are considered to be potential proinflammatory adipocytokines (50). Under current experimental conditions, the regulation of genes involved in the cellular response to lipids might suggest the modulation of lipid-related intracellular signaling pathways in the fish fed the EOs-supplemented diet, with a potential role on the immune-inflammatory profile obtained.

Lipids affect the gut microbiota both as substrates for bacterial metabolic processes and by inhibiting bacterial growth by toxic influence (51). In turn, gut microbiota are also pointed as one of the key elements affecting inflammation associated with lipid metabolism dysfunction (2). In fish, the gut microbiota are also recognized to affect considerably the lipid metabolism of the host (41). In agreement to our transcriptional analysis and to the abovementioned findings, the PICRUSt analysis of the microbiota from the anterior intestine of fish fed the EOs-supplemented diet showed a higher abundance of sequences associated with lipid metabolism when compared to the control group. In fact, garlic and its derivatives are widely recognized for their hypolipidemic effect. For instance, one of the primary components of garlic, diallyl disulfide, was suggested to affect both lipid metabolism and gut microbiota in mice through the regulation of the expression of genes associated with lipogenesis and lipid metabolism (52). In other studies, the combined dietary administration of thymol and carvacrol have demonstrated to modulate the intestinal microbiota in piglets, changes that were correlated with an increase in lipid metabolism, among others metabolic effects (53). In this context, our taxonomical analysis at the genus level showed a significant increase in the abundance of *Corynebacterium* (Actinobacteria) in the anterior intestine of fish fed the EOs-supplemented diet. This genus has been reported as a predominant one along the whole digestive tract of gilthead seabream, while its abundance may be modulated by functional diets (54) and dietary lipid levels (55). These results are of special relevance since *Corynebacterium* species are reputed for contributing to manganese acquisition and producing superoxide dismutase and lipases to form organic fatty acids and thioalcohols (56). This genus also showed a higher presence in rainbow trout (*Oncorhynchus mykiss*) intestinal microbiota when the fish were fed high lipid diets (55), evidencing the impact of the tested EOs on the host and microbial lipid metabolism.

Furthermore, a decrease in the abundance of *Rothia* was also detected in the posterior intestine of fish fed the EOs-supplemented diet. *Rothia* abundance was observed to be affected by fish age and sex hormones in gilthead sea bream (14). In effect, the results from our transcriptional analysis revealed a positive regulation of processes related with a response to hormone stimulus. Changes in hormone secretion, such as cortisol, may interfere with the gut immune response (57) and microbiome (58), which could explain the obtained immunity activation and regulation of hormone-sensitive bacteria, such as those belonging to *Rothia* (14). Moreover, stress and stress-related hormones are known to affect carbohydrate, protein, and lipid metabolisms in fish (59), which in turn are also regulated in the host by the gut microbiota. In this sense, a similar feed additive containing garlic and labiatae plant EOs (0.02% inclusion) was demonstrated to reduce significantly plasma cortisol levels in European seabass (*Dicentrarchus labrax*) (60); thus, the potential regulation of stress-related hormones by the tested EOs could explain the response to steroid hormones processes obtained in our functional analysis.

In the present study, the administration of the garlic, carvacrol, and thymol EOs positively affected the expression of growth hormone 2 (*gh2*), although no significant differences in somatic growth were observed at the end of the 65 days of feeding trial. However, GH is not only involved in somatic growth; this hormone also directly stimulates several fish immune factors (61), and participates in the epithelial osmoregulation of euryhaline fish, interacting with cortisol to increase secretory chloride cells and ion transporters involved in salt secretion, such Na^+^/K^+^ ATPase (62). As a matter of fact, besides immunity and digestion, the gastrointestinal tract of marine teleost fish also plays an important role in osmoregulation. Under this context, the carbonic anhydrase 2 (*ca2*) was the second most positively affected gene by the EOs inclusion in the diet, playing an active role in acid–base regulation through bicarbonate secretion and facilitating epithelial water transport (63). In fact, osmoregulation has been linked to endocrine secretory factors with a significant impact on the fish immune system and microbiota (64).

As previously referred, the *Bacteroidia* class (Bacteroidetes) decreased significantly in the posterior intestine of fish fed the EOs diet. Kan et al. (65) demonstrated that within the *Bacteroidia* class, *Bacteroides* genus abundance increased in goldfish (*Carassius auratus*) when exposed to a toxic environment. Interestingly, our microbiota analysis showed a higher abundance of bacterial 16S rRNA sequences associated with the metabolism of glutathione in the anterior intestine. Glutathione is one of the most important intracellular antioxidant and antitoxin enzymes, whereas its metabolism is regulated by the gut microbiota through the modulation of the amino acid metabolism of the host (66) and tissue oxidative stress (67). Furthermore, glutathione plays important roles in nutrient metabolism and in the regulation of cellular events, such as gene expression, DNA and protein synthesis, cell proliferation and apoptosis, immune response, among others (67). Glutathione S-transferase is one of the key enzymes involved in the second phase of xenobiotics' metabolism and cellular detoxification, catalyzing the conjugation of reduced glutathione to various substances; thus, suggesting a key role in the host immune response modulation (68). Accordingly, in our transcriptional analysis, the glutathione S-transferase theta 2B (*gstt2b*) gene was observed to be upregulated in the intestine of gilthead seabream following the administration of dietary EOs. The differences observed regarding both transcriptional and microbiota analysis between our experimental diets suggested an improvement of the enterocytes' lipid metabolism and detoxification potential promoted by the additive.

The microbiota analysis also showed a reduction in the proportion of bacterial sequences related to drug metabolism. Accordingly, the EOs-supplemented diet promoted the increase of the cytochrome P450 2J2 (*cyp2j2*) gene transcripts in the intestine of the gilthead seabream. In fish, the cytochrome P450 proteins, and CYP2 family members, in particular, participate in the metabolism of steroidal hormones and other lipids, besides their role in the metabolism of exogenous compounds like drugs and pharmaceuticals (69). Several garlic organosulfur compounds, as well as carvacrol, have been described to selectively modulate the levels of cytochrome P450 genes and proteins (70, 71). Moreover, the mitochondrial peroxiredoxin 3 (*prdx3*) and cathepsin B (*ctsb*) genes were downregulated. Both *prdx3* and *ctsb* are biomarkers of fish stressors (72), whose downregulation might indicate a decrease of the oxidative stress in the fish intestine and a positive impact of the tested additive on fish welfare. Overall, our results indicate that the administrated EOs promotes the enhancement of the antioxidative status in the fish intestine, supporting the gut homeostasis under an immune stimulation scenario.

### Effect of Garlic, Carvacrol, and Thymol EOs Additive on the Response to Organic Nitrogen and Aromatic Compounds

In our transcriptional analysis, several genes comprising a response to nitrogenous compounds related processes were also observed to be positively regulated by the presence of garlic, carvacrol, and thymol EOs in the diet. Interestingly, the inclusion of the EOs in the gilthead seabream diet showed a significant decrease in the abundance of the genera *Paracoccus* (Proteobacteria), *Prevotella* (Bacteroidetes) in the posterior intestine, and *Comamonas* (Proteobacteria) in the anterior intestine of fish. All these bacteria are reputed for their capacity for nitrate reduction, as well as being potentially involved in the metabolism of nitrogenous compounds (73). In particular, *Prevotella*, are members of the anaerobic, hydrogen sulfide producing bacterial community (73) that have been previously detected in the intestine of gilthead seabream (54). In humans, an increase in *Pretovella* species at mucosal sites is often associated with chronic inflammation (74). In our study, the PICRUSt analysis showed a lower abundance of predicted carbohydrate degradation pathway in the anterior intestine of fish fed the EOs-supplemented diet, which may be associated with a reduction in *Prevotella* abundance. *Paracoccus* is a genus in the family *Rhodobacteraceae* previously reported in gilthead seabream gut and described as a potential probiotic for this species (75). The relevance of the decrease in the abundance of *Paracoccus* genus needs further investigations in terms of its impact on the condition of the host as no negative effects on gut conditions were observed under present nutritional conditions.

Furthermore, some *Comamonas* strains are also known to have genes for naphthalene degradation (76). The posterior intestine of fish fed the EOs-supplemented diet showed an increase in bacterial sequences related to naphthalene degradation. Naphthalene is an aromatic hydrocarbon present in many EOs with antibacterial, antioxidant, and antiparasitic properties (77). Although suggested to have a positive impact at low concentrations by decreasing DNA damage in some fish species (78), an enhancement in naphthalene and similar compounds degradation is crucial in order to avoid a potential toxicity of the EOs for the host.

The transcriptional analysis showed the positive regulation of the response to alkaloids biological process in the fish fed the EOs-supplemented diet. Alkaloids are versatile heterocyclic nitrogen compounds produced by plants, that along with EOs and phenolic compounds, provide antipathogenic and antioxidant protection (79). This response may not only be associated with the previously referred alteration in the metabolism of nitrogen and carbohydrates induced by the microbiota reshaping, but also with the direct response of the intestinal mucosa to the phenolic monoterpenes carvacrol and thymol (80) and other cyclic compounds derived from garlic (81) with recognized immunomodulatory properties. Moreover, allicin, the main antimicrobial compound in garlic, is also a sulfoxide that bacteria can use in the sulfur-relay system (82). This is in agreement with the observed increase in sequences associated with genes of the sulfur-relay system in the posterior intestine of fish fed the EOs-supplemented diet. Thereafter, considering the complexity of the EOs biochemistry, the transcriptional and bacterial response to those compounds is equally multifaceted. Further studies should be addressed in order to clarify the impact of these potential metabolic alterations in the gilthead seabream gut immune status.

### Effect of Garlic, Carvacrol, and Thymol EOs Additive on Symbiosis Processes

The intricate host-microbiota symbiosis in the fish is still substantially unexplored when compared with mammals, and considering its complex challenges to define an “ideal” microbiome for each species since microbiota are strongly modulated by environmental and dietary factors (2). Even though both transcriptional and microbiota modulations by the EOs supplementation were observed, our results fit within the farmed gilthead seabream gut microbiome profile in terms of dominant phyla bacterial composition (14), discarding warnings of a diet-induced dysbiosis. The transcriptomic functional analysis was able to particularly detect such interactions through the expression of several genes related to symbiotic, multi-organism processes, and interspecies interaction between organisms.

For instance, the microbiota taxonomical analysis at the genus level showed an increase in the abundance of *Photobacterium* (Proteobacteria, Vibrionaceae) in the anterior intestine of fish fed the EOs diet. Although some members of this genus, such as *Photobacterium damselae* subsp *piscicida* and *P. damselae* subsp *damselae* have been reported as important pathogens for gilthead seabream (83), they are generally detected in the intestine of healthy specimens (84, 85). Most species of the *Photobacterium* genus are non-pathogenic and are usually in a symbiotic relationship with marine organisms as enteric commensals. In fact, *Photobacterium* spp. have been even found to be beneficial as a member of the fish intestinal microbiota by its ability to aid with digestion of compounds, such as chitin (86), to produce polyunsaturated fatty acids or even antibacterial secondary metabolites that could inhibit the growth of other pathogenic bacteria (87). This genus has been reported as a member of the intestinal microbiota of marine farmed fish, including gilthead seabream (54, 85), and it has been demonstrated that this genus is one of the most modulated genera in the fish when applying functional diets (88). Regarding the antimicrobial effect of the EOs-supplemented diet, an *in vitro* study demonstrated that the ethanolic extracts of oregano leaves, predominantly composed of carvacrol and thymol, presented a strong bactericidal activity against several pathogens including *Photobacterium damselae*, besides its immunostimulatory effect on gilthead seabream head kidney leukocytes (89). Therefore, our results might suggest a selective antimicrobial effect of the compounds administrated, evidencing the importance of the host-microbiota symbiotic relationship in the modulation of the response to a dietary change.

Additionally, in our transcriptional analysis, the retinoic acid receptor alpha (*rara*) and the retinoic X receptor beta (*rxrb*) genes were both up and downregulated, respectively, in the gut of fish fed the EOs-supplemented diet. The retinoic acid (RA) is the most important transcriptionally active component of the vitamin A, an essential dietary nutrient for fish that plays a significant role in a range of physiological processes including the differentiation and maintenance of epithelial cells and immunity (90). Under this context, another case of symbiotic interaction between organisms is the relation between vitamin A metabolism of the host and its commensal microbiota. Remarkably, *Clostridia* (Firmicutes) abundance was significantly reduced in gilthead seabream fed the EOs diet, which could then be positively affecting the RA availability and the observed regulation of the nuclear receptors (90), potentially participating in the local immunity boost observed in our study. In fact, dietary garlic powder was demonstrated to have an antimicrobial effect on *Clostridium* human bacteria, being suggested to temporarily modulate the gut microbiota (91). In rainbow trout, different levels of garlic extract (1%, 1.5%, and 2%) positively affected the abundance of this genus (4). Curiously, a similar dietary additive composed of garlic and labiatae plants oils was observed to enrich the *Clostridia* class in European seabass fed a low fishmeal and fish oil diet (30). However, carvacrol and thymol, in particular, were numerously observed to exert an antimicrobial effect on *Clostridium* species, proving beneficial for the gut health of several organisms (92); thus, attributing to carvacrol and thymol the main role in the observed reduction of the genus. Given the significance of this symbiosis, the manipulation of RA signaling derived from dietary components acting directly on nuclear receptors and/or on the intestinal microbiota might represent a strategy to promote gut immunostimulation.

### Effect of Garlic, Carvacrol, and Thymol EOs Additive on Gene Expression and RNA Processing

Dietary manipulations are widely recognized to directly or indirectly influence the regulation of the fish gut gene expression, in order to reshape its metabolic and physiological responses to different requirements. Indeed, the utmost upregulated biological processes in the intestine of fish fed the functional feed additive tested in our study, in terms of the number of DEGs, were those related to gene expression and processes involved in RNA processing, RNA splicing, mRNA metabolism, and mRNA and ribonucleoprotein export from nucleus. The regulation of gene expression comprises diverse cell mechanisms in order to increase or decrease the production of a specific gene product, either RNA or a protein. For instance, several zinc finger proteins were up (*znf572, zeb2, znf74, zc3h11a*, and *znf214*) and down (*znf133, znf551*) regulated in our transcriptional analysis. Besides the stimulation of the transcriptional machinery (93), several genes involved in the spliceosome-mediated splicing (*snrnp200, sart1, hnrnpu*, and *prpf8*) were also observed to be upregulated by dietary EOs. The spliceosome splicing complex removes intronic non-coding sequences from pre-mRNA to form mature mRNA that can be translated into protein (94).

In another hand, the intestine is *per se* a highly regenerative organ characterized by its continual cell renewal, allowing the epithelium to bear the constant exertion of food digestion, nutrient absorption, and waste elimination (6). Either tissue damage or microbial invasion promotes inflammation and possible DNA damage, so its repair plays a vital role in maintaining genomic integrity during the cell cycle. For instance, DNA damage responses may be induced by proinflammatory cytokines (95), in which transcriptional response appeared not to be promoted by the EOs in our study, as previously discussed. However, genes coding DNA damage checkpoint proteins were up (*fbxo31, gltscr2, wisp1, usp10*, and *cdk5rap3*) and down (*nbn*) regulated by the EOs-supplemented diet, evidencing a regulation of the cell turnover independent from inflammatory stimuli. This hypothesis is reinforced by the upregulation of *cdk5rap3*, the gene encoding CDK5 regulatory subunit associated protein 3, an interactor controlling cell proliferation that among other functions negatively regulates NF-kB mediated gene transcription (96), as initially suggested. Our results also evidence the tight functional connection and coordination between DNA damage responses and immunity, a link that is recognized by its involvement in the protection of the host from infectious microorganisms and surveillance against malignant diseases (97). Therefore, the upregulation of a substantial number of genes that modulates others' expression and that has an implication in transcriptional, translational and DNA repair processes validates the effect of the EOs-supplemented diet on the direct transcriptional regulation of several intestinal cellular processes, including the modulation of the inflammatory and immune response.

## Conclusions

The present complementary analysis of the intestinal transcriptomic profiling and microbiota response to a diet supplemented with garlic, carvacrol, and thymol EOs aimed to take a further step in the evaluation of functional feeds in an attempt to understand how diet-induced shifts can affect the overall gut status of farmed fish from an integrative perspective. This kind of integrative analysis can lead to the “chicken or egg” causality dilemma, and exact mechanisms are still elusive. Nevertheless, the present work suggested that the dietary administration of garlic, carvacrol, and thymol EOs modulated the immune transcriptional response of the mid-anterior intestinal mucosa *per se*, but also its microbiota composition, resulting in complex interactions that resulted in the activation of significant biological processes. Taken together, the combined regulation of the referred pathways could suggest the promotion of an immune reinforcement by the EOs dietary administration *in situ*, most probably induced by host-microbial co-metabolism, which could further attenuate the processes of pathogenesis, putting in evidence the re-adaptation response of the intestinal mucosa to the changes observed in the microbiota composition, and *vice versa*. Moreover, no indications of an inflammation associated with the immunostimulation, which could compromise the intestine integrity, were observed. Since no interference with fish growth was observed, promoted changes in both the intestine mucosa and microbiota were assumed to not significantly affect the gut overall metabolism and nutritional status. Thus, the use of the tested EOs is suggested as a promising alternative to chemotherapeutics to be further evaluated in functional diets under the presence of biotic or abiotic stressors.

## Data Availability Statement

The datasets presented in this study can be found in online repositories. The names of the repository/repositories and accession number(s) can be found in the article/Supplementary Material.

## Ethics Statement

All animal experimental procedures were conducted in compliance with the research protocol approved by the IRTA's Committee of Ethics and Animal Experimentation and in accordance with the Guidelines of the European Union Council (86/609/EU) for the use of laboratory animals.

## Author Contributions

AE and EG designed and carried out the experiments. Biological samplings were performed by EG, AE, RS, and JF. The transcriptomic data analysis and interpretation were performed by JF, RS, EV-V, and FER-L. MB, IC, and MM carried out the microbiota analyses. YR-C reviewed and validated the methodology used in the study. The study was supervised by EG, FER-L, and LT. JF wrote the original draft. All the authors provided the critical feedback, read, and agreed to the published version of the manuscript.

## Conflict of Interest

JF is a current TECNOVIT-FARMFAES S.L. employee conducting an Industrial Ph.D. The remaining authors declare that the research was conducted in the absence of any commercial or financial relationships that could be construed as a potential conflict of interest.

## References

[1] Asif MB Hai FI Price WE Nghiem LD. Impact of pharmaceutically active compounds in marine environment on aquaculture. In: Hai F Visvanathan C Boopathy R, editors. Sustainable Aquaculture. Applied Environmental Science and Engineering for a Sustainable Future. Cham: Springer (2018) p. 265–99.

[2] Hoseinifar SH Van Doan H Dadar M Ringø E Harikrishnan R. Feed additives, gut microbiota, and health in finfish aquaculture. In: Derome N, editor. Microbial Communities in Aquaculture Ecosystems: Improving Productivity and Sustainability. Cham: Springer International Publishing (2019). p. 121–42.

[3] Sutili FJ Gatlin DM Heinzmann BM Baldisserotto B. Plant essential oils as fish diet additives: benefits on fish health and stability in feed. Rev Aquac. (2017) 10:716–26. 10.1111/raq.12197

[4] Büyükdeveci ME Balcázar JL Demirkale I Dikel S. Effects of garlic-supplemented diet on growth performance and intestinal microbiota of rainbow trout (*Oncorhynchus mykiss*). Aquaculture. (2018) 486:170–4. 10.1016/j.aquaculture.2017.12.022

[5] Zhang R Wang XW Liu LL Cao YC Zhu H. Dietary oregano essential oil improved the immune response, activity of digestive enzymes, and intestinal microbiota of the koi carp, *Cyprinus carpio*. Aquaculture. (2020) 518:734781. 10.1016/j.aquaculture.2019.734781

[6] Celi P Cowieson AJ Fru-Nji F Steinert RE Kluenter AM Verlhac V. Gastrointestinal functionality in animal nutrition and health: new opportunities for sustainable animal production. Anim Feed Sci Technol. (2017) 234:88–100. 10.1016/j.anifeedsci.2017.09.012

[7] Salinas I Parra D. 6–fish mucosal immunity: intestine. In: Beck BH Peatman E, editor. Mucosal Health in Aquaculture. San Diego, CA: Academic Press (2015). p. 135–70.

[8] Nadal AL Ikeda-Ohtsubo W Sipkema D Peggs D McGurk C Forlenza M. Feed, microbiota, and gut immunity: using the zebrafish model to understand fish health. Front Immunol. (2020) 11:114. 10.3389/fimmu.2020.00114 32117265 PMC7014991

[9] Firmino JP Vallejos-Vidal E Sarasquete C Ortiz-Delgado JB Balasch JC Tort L. Unveiling the effect of dietary essential oils supplementation in *Sparus aurata* gills and its efficiency against the infestation by *Sparicotyle chrysophrii*. Sci Rep. (2020) 10:17764. 10.1038/s41598-020-74625-5 33082387 PMC7576129

[10] Dezfooli SM Gutierrez-Maddox N Alfaro A Seyfoddin A. Encapsulation for delivering bioactives in aquaculture. Rev Aquac. (2019) 11:631–60. 10.1111/raq.12250

[11] Sutton SG Bult TP Haedrich RL. Relationships among fat weight, body weight, water weight, and condition factors in wild atlantic salmon parr. Trans Am Fish Soc. (2000) 129:527–38. 10.1577/1548-8659(2000)129<0527:RAFWBW>2.0.CO;2

[12] Calduch-Giner JA Sitjà-Bobadilla A Pérez-Sánchez J. Gene expression profiling reveals functional specialization along the intestinal tract of a carnivorous teleostean fish (*Dicentrarchus labrax*). Front Physiol. (2016) 7:359. 10.3389/fphys.2016.00359 27610085 PMC4997091

[13] Tapia-Paniagua ST Chabrillón M Díaz-Rosales P de la Banda IG Lobo C Balebona MC. Intestinal microbiota diversity of the flat fish *Solea senegalensis* (Kaup, 1858) following probiotic administration. Microb Ecol. (2010) 60:310–9. 10.1007/s00248-010-9680-z 20556376

[14] Piazzon MC Naya-Català F Simó-Mirabet P Picard-Sánchez A Roig FJ Calduch-Giner JA. Sex, age, and bacteria: how the intestinal microbiota is modulated in a protandrous hermaphrodite fish. Front Microbiol. 10:2512. 10.3389/fmicb.2019.02512 31736931 PMC6834695

[15] Glickman MH Ciechanover A. The ubiquitin-proteasome proteolytic pathway: destruction for the sake of construction. Physiol Rev. (2002) 82:373–428. 10.1152/physrev.00027.2001 11917093

[16] Dorrington MG Fraser IDC. NF-κB signaling in macrophages: dynamics, crosstalk, and signal integration. Front Immunol. (2019) 10:705. 10.3389/fimmu.2019.00705 31024544 PMC6465568

[17] Neish AS Gewirtz AT Zeng H Young AN Hobert ME Karmali V. Prokaryotic regulation of epithelial responses by inhibition of IkappaB-alpha ubiquitination. Science. (2000) 289:1560–3. 10.1126/science.289.5484.1560 10968793

[18] Nya EJ Dawood Z Austin B. The garlic component, allicin, prevents disease caused by *Aeromonas hydrophila* in rainbow trout, *Oncorhynchus mykiss* (Walbaum). J Fish Dis. (2010) 33:293–300. 10.1111/j.1365-2761.2009.01121.x 20082660

[19] Ahmadifar E Razeghi Mansour M Keramat Amirkolaie A Fadaii Rayeni M. Growth efficiency, survival and haematological changes in great sturgeon (*Huso huso* Linnaeus, 1758) juveniles fed diets supplemented with different levels of thymol–carvacrol. Anim Feed Sci Technol. (2014) 198:304–8. 10.1016/j.anifeedsci.2014.08.012

[20] Welch HC. Regulation and function of P-Rex family Rac-GEFs. Small GTPases. (2015) 6:49–70. 10.4161/21541248.2014.973770 25961466 PMC4601503

[21] Baumann M Pham CT Benarafa C. SerpinB1 is critical for neutrophil survival through cell-autonomous inhibition of cathepsin G. Blood. (2013) 121:3900–7. 10.1182/blood-2012-09-455022 23532733 PMC3650706

[22] Choi YJ Kim S Choi Y Nielsen TB Yan J Lu A. SERPINB1-mediated checkpoint of inflammatory caspase activation. Nat Immunol. (2019) 20:276–87. 10.1038/s41590-018-0303-z 30692621 PMC6450391

[23] Spicer BA Conroy PJ Law RHP Voskoboinik I Whisstock JC. Perforin-A key (shaped) weapon in the immunological arsenal. Semin Cell Dev Biol. (2017) 72:117–23. 10.1016/j.semcdb.2017.07.033 28757431

[24] Leal E Ordás MC Soleto I Zarza C McGurk C Tafalla C. Functional nutrition modulates the early immune response against viral haemorrhagic septicaemia virus (VHSV) in rainbow trout. Fish Shellfish Immunol. (2019) 94:769–79. 10.1016/j.fsi.2019.09.070 31580935

[25] Ginés S Mariño M Mallol J Canela EI Morimoto C Callebaut C. Regulation of epithelial and lymphocyte cell adhesion by adenosine deaminase-CD26 interaction. Biochem J. (2002) 361:203–9. 10.1042/0264-6021:3610203 11772392 PMC1222300

[26] Casanova V Naval-Macabuhay I Massanella M Rodríguez-García M Blanco J Gatell JM. Adenosine deaminase enhances the immunogenicity of human dendritic cells from healthy and HIV-infected individuals. PLoS ONE. (2012) 7:e51287. 10.1371/journal.pone.0051287 23240012 PMC3519778

[27] Saiz ML Rocha-Perugini V Sánchez-Madrid F. Tetraspanins as organizers of antigen-presenting cell function. Front Immunol. (2018) 9:1074. 10.3389/fimmu.2018.01074 29875769 PMC5974036

[28] Peñaranda MMD Jensen I Tollersrud LG Bruun JA Jørgensen JB. Profiling the Atlantic salmon IgM^+^ B cell surface proteome: novel information on teleost fish B cell protein repertoire and identification of potential B cell markers. Front Immunol. (2019) 10:37. 10.3389/fimmu.2019.00037 30761128 PMC6362898

[29] Green LR Monk PN Partridge LJ Morris P Gorringe AR Read RC. Cooperative role for tetraspanins in adhesin-mediated attachment of bacterial species to human epithelial cells. Infect Immun. (2011) 79:2241–9. 10.1128/iai.01354-10 21464080 PMC3125835

[30] Rimoldi S Torrecillas S Montero D Gini E Makol A Valdenegro VV. Assessment of dietary supplementation with galactomannan oligosaccharides and phytogenics on gut microbiota of European sea bass (*Dicentrarchus Labrax*) fed low fishmeal and fish oil based diet. PLoS ONE. (2020) 15:e0231494. 10.1371/journal.pone.0231494 32298317 PMC7162502

[31] Torrecillas S Terova G Makol A Serradell A Valdenegro V Gini E. Dietary phytogenics and galactomannan oligosaccharides in low fish meal and fish oil-based diets for European sea bass (*Dicentrarchus labrax*) juveniles: Effects on gut health and implications on *in vivo* gut bacterial translocation. PLoS ONE. (2019) 14:e0222063. 10.1371/journal.pone.0222063 31532807 PMC6750610

[32] Hampson DJ Ahmed N. Spirochaetes as intestinal pathogens: lessons from a *Brachyspira* genome. Gut Pathogens. (2009) 1:10. 10.1186/1757-4749-1-10 19405984 PMC2680911

[33] Prashar A Schnettger L Bernard EM Gutierrez MG. Rab GTPases in immunity and inflammation. Front Cell Infect Microbiol. (2017) 7:435. 10.3389/fcimb.2017.00435 29034219 PMC5627064

[34] Bonello S Zähringer C BelAiba RS Djordjevic T Hess J Michiels C. Reactive oxygen species activate the HIF-1alpha promoter via a functional NFkappaB site. Arterioscler Thromb Vasc Biol. (2007) 27:755–61. 10.1161/01.ATV.0000258979.92828.bc 17272744

[35] Sveinbjornsson B Olsen R Paulsen S. Immunocytochemical localization of lysozyme in intestinal eosinophilic granule cells (EGCs) of Atlantic salmon, *Salmo salar* L. J Fish Dis. (1996) 19:349–55. 10.1046/j.1365-2761.1996.d01-87.x

[36] Szmola R Kukor Z Sahin-Tóth M. Human mesotrypsin is a unique digestive protease specialized for the degradation of trypsin inhibitors. J Biol Chem. (2003) 278:48580–9. 10.1074/jbc.M310301200 14507909 PMC1393292

[37] Kokou F Fountoulaki E. Aquaculture waste production associated with antinutrient presence in common fish feed plant ingredients. Aquaculture. (2018) 495:295–310. 10.1016/j.aquaculture.2018.06.003

[38] Perez-Sanchez J Benedito-Palos L Estensoro I Petropoulos Y Calduch-Giner JA Browdy CL. Effects of dietary NEXT ENHANCE®150 on growth performance and expression of immune and intestinal integrity related genes in gilthead sea bream (*Sparus aurata* L.). Fish Shellfish Immunol. (2015) 44:117–28. 10.1016/j.fsi.2015.01.039 25681752

[39] Calduch-Giner JA Sitjà-Bobadilla A Davey GC Cairns MT Kaushik S Pérez-Sánchez J. Dietary vegetable oils do not alter the intestine transcriptome of gilthead sea bream (*Sparus aurata*), but modulate the transcriptomic response to infection with *Enteromyxum leei*. BMC Genomics. (2012) 13:470. 10.1186/1471-2164-13-470 22967181 PMC3444936

[40] Dawson PA Karpen SJ. Intestinal transport and metabolism of bile acids. J Lipid Res. (2015) 56:1085–99. 10.1194/jlr.R054114 25210150 PMC4442867

[41] Sheng Y Ren H Limbu SM Sun Y Qiao F Zhai W. The presence or absence of intestinal microbiota affects lipid deposition and related genes expression in zebrafish (*Danio rerio*). Front Microbiol. (2018) 9:1124. 10.3389/fmicb.2018.01124 29896183 PMC5987169

[42] Campbell C McKenney PT Konstantinovsky D Isaeva O Schizas M Verter J. Bacterial metabolism of bile acids promotes generation of peripheral regulatory T cells. Nature. (2020) 581:475–9. 10.1038/s41586-020-2193-0 32461639 PMC7540721

[43] Allers K Stahl-Hennig C Fiedler T Wibberg D Hofmann J Kunkel D. The colonic mucosa-associated microbiome in SIV infection: shift towards bacteroidetes coincides with mucosal CD4^+^ T cell depletion and enterocyte damage. Sci Rep. (2020) 10:10887. 10.1038/s41598-020-67843-4 32616803 PMC7331662

[44] Fiorucci S Biagioli M Zampella A Distrutti E. Bile acids activated receptors regulate innate immunity. Front Immunol. (2018) 9:1853. 10.3389/fimmu.2018.01853 30150987 PMC6099188

[45] Spanogiannopoulos P Bess EN Carmody RN Turnbaugh PJ. The microbial pharmacists within us: a metagenomic view of xenobiotic metabolism. Nat Rev Microbiol. (2016) 14:273–87. 10.1038/nrmicro.2016.17 26972811 PMC5243131

[46] Wang S-Z Yu Y-J Adeli K. Role of gut microbiota in neuroendocrine regulation of carbohydrate and lipid metabolism via the microbiota-gut-brain-liver axis. Microorganisms. (2020) 8:527. 10.3390/microorganisms8040527 32272588 PMC7232453

[47] Diaz M Dopido R Gomez T Rodriguez C. Membrane lipid microenvironment modulates thermodynamic properties of the Na^+^-K^+^-ATPase in branchial and intestinal epithelia in euryhaline fish *in vivo*. Front Physiol. (2016) 7:589. 10.3389/fphys.2016.00589 28018232 PMC5156835

[48] Niespolo C Salamanca Viloria J Deshmukh S Villacanas Perez O Sudbery I Wilson H. BS25 Investigating the MIR-101-3P/TRIB1 axis in macrophage immunometabolism. Heart. (2019) 105:A156. 10.1136/heartjnl-2019-BCS.188

[49] Berk PD Zhou S Kiang C Stump DD Fan X Bradbury MW. Selective upregulation of fatty acid uptake by adipocytes characterizes both genetic and diet-induced obesity in rodents. J Biol Chem. (1999) 274:28626–31. 10.1074/jbc.274.40.28626 10497230

[50] Sanz Y. Moya-Pérez A. Microbiota, Inflammation and obesity. In: Lyte M. Cryan J. editors. Microbial Endocrinology: The Microbiota-Gut-Brain Axis in Health and Disease. Advances in Experimental Medicine and Biology, New York, NY: Springer (2014) 817. 10.1007/978-1-4939-0897-4_14 24997040

[51] Schoeler M Caesar R. Dietary lipids, gut microbiota and lipid metabolism. Rev Endocr Metab Disord. (2019) 20:461–72. 10.1007/s11154-019-09512-0 31707624 PMC6938793

[52] Yang Y Yang F Huang M Wu H Yang C Zhang X. Fatty liver and alteration of the gut microbiome induced by diallyl disulfide. Int J Mol Med. (2019) 44:1908–20. 10.3892/ijmm.2019.4350 31573042 PMC6777666

[53] Li Y Fu X Ma X Geng S Jiang X Huang Q. Intestinal microbiome-metabolome responses to essential oils in piglets. Front Microbiol. (2018) 9:1988. 10.3389/fmicb.2018.01988 30210470 PMC6120982

[54] Estruch G Collado MC Peñaranda DS Tomás Vidal A Jover Cerdá M Pérez Martínez G Martinez-Llorens S. Impact of fishmeal replacement in diets for gilthead sea bream (*Sparus aurata*) on the gastrointestinal microbiota determined by pyrosequencing the 16S rRNA gene. PLoS ONE. (2015) 10:e0136389. 10.1371/journal.pone.0136389 26317431 PMC4552794

[55] Huyben D Vidaković A Werner Hallgren S Langeland M. High-throughput sequencing of gut microbiota in rainbow trout (*Oncorhynchus mykiss*) fed larval and pre-pupae stages of black soldier fly (*Hermetia illucens*). Aquaculture. (2019) 500:485–91. 10.1016/j.aquaculture.2018.10.034

[56] Johnson DI. *Corynebacterium* spp. In: Johnson DI, editor. Bacterial Pathogens and Their Virulence Factors. Cham: Springer International Publishing (2018). p. 73–9.

[57] Kvamme BO Gadan K Finne-Fridell F Niklasson L Sundh H Sundell K. Modulation of innate immune responses in Atlantic salmon by chronic hypoxia-induced stress. Fish Shellfish Immunol. (2013) 34:55–65. 10.1016/j.fsi.2012.10.006 23085636

[58] Webster TMU Rodriguez-Barreto D Consuegra S Garcia de Leaniz C. Cortisol-induced signatures of stress in the fish microbiome. bioRxiv. (2019) 826503. 10.1101/826503 PMC7381252 32765459

[59] Mommsen TP Vijayan MM Moon TW. Cortisol in teleosts: dynamics, mechanisms of action, and metabolic regulation. Rev Fish Biol Fish. (1999) 9:211–68. 10.1023/A:1008924418720

[60] Serradell A Torrecillas S Makol A Valdenegro V Fernández-Montero A Acosta F. Prebiotics and phytogenics functional additives in low fish meal and fish oil based diets for European sea bass (*Dicentrarchus labrax*): Effects on stress and immune responses. Fish Shellfish Immunol. (2020) 100:219–29. 10.1016/j.fsi.2020.03.016 32160965

[61] Yada T. Growth hormone and fish immune system. Gen Compar Endocrinol. (2007) 152:353–8. 10.1016/j.ygcen.2007.01.045 17382328

[62] McCormick SD. Endocrine control of osmoregulation in teleost fish. Am Zool. (2015) 41:781–94. 10.1093/icb/41.4.781

[63] Lin TY Liao BK Horng JL Yan JJ Hsiao CD Hwang PP. Carbonic anhydrase 2-like a and 15a are involved in acid-base regulation and Na^+^ uptake in zebrafish H^+^-ATPase-rich cells. Am J Physiol Cell Physiol. (2008) 294:C1250–60. 10.1152/ajpcell.00021.2008 18322140

[64] Lin G Zheng M Li S Xie J Fang W Gao D. Response of gut microbiota and immune function to hypoosmotic stress in the yellowfin seabream (*Acanthopagrus latus*). Sci Total Environ. (2020) 745:140976. 10.1016/j.scitotenv.2020.140976 32736105

[65] Kan H Zhao F Zhang XX Ren H Gao S. Correlations of gut microbial community shift with hepatic damage and growth inhibition of *Carassius auratus* induced by pentachlorophenol exposure. Environ Sci Technol. (2015) 49:11894–902. 10.1021/acs.est.5b02990 26378342

[66] Mardinoglu A Shoaie S Bergentall M Ghaffari P Zhang C Larsson E. The gut microbiota modulates host amino acid and glutathione metabolism in mice. Mol Syst Biol. (2015) 11:834. 10.15252/msb.20156487 26475342 PMC4631205

[67] Wu G Fang YZ Yang S Lupton JR Turner ND. Glutathione metabolism and its implications for health. J Nutr. (2004) 134:489–92. 10.1093/jn/134.3.489 14988435

[68] Vallejos-Vidal E Reyes-Cerpa S Rivas-Pardo JA Maisey K Yáñez JM Valenzuela H. Single-nucleotide polymorphisms (SNP) mining and their effect on the tridimensional protein structure prediction in a set of immunity-related expressed sequence tags (EST) in Atlantic salmon (*Salmo salar*). Front Genet. (2020) 10:1406. 10.3389/fgene.2019.01406 32174954 PMC7056891

[69] Schlenk D Celander M Gallagher E George S James M Kullman S. Biotransformation in fishes. In: Di Giulio RT Hinton DE, editors. The Toxicology of Fishes. Boca Raton, FL: CRC Press (2008). p. 153–234.

[70] Gao C Jiang X Wang H Zhao Z Wang W. Drug metabolism and pharmacokinetics of organosulfur compounds from garlic. J Drug Metab Toxicol. (2013) 4:159. 10.4172/2157-7609.100015920930421

[71] Khan I Bhardwaj M Shukla S Min SH Choi DK Bajpai VK. Carvacrol inhibits cytochrome P450 and protects against binge alcohol-induced liver toxicity. Food Chem Toxicol. (2019) 131:110582. 10.1016/j.fct.2019.110582 31220535

[72] Espinosa C Cuesta A Esteban M. Effects of dietary polyvinylchloride microparticles on general health, immune status and expression of several genes related to stress in gilthead seabream (*Sparus aurata* L.). Fish Shellfish Immunol. (2017) 68:251–9. 10.1016/j.fsi.2017.07.006 28684324

[73] Purushe J Fouts DE Morrison M White BA Mackie RI Coutinho PM. Comparative genome analysis of *Prevotella ruminicola* and *Prevotella bryantii*: insights into their environmental niche. Microb Ecol. (2010) 60:721–9. 10.1007/s00248-010-9692-8 20585943

[74] Larsen JM. The immune response to *Prevotella* bacteria in chronic inflammatory disease. Immunology. (2017) 151:363–74. 10.1111/imm.12760 28542929 PMC5506432

[75] Makridis P Martins S Vercauteren T Van Driessche K Decamp O Dinis MT. Evaluation of candidate probiotic strains for gilthead sea bream larvae (*Sparus aurata*) using an *in vivo* approach. Lett Appl Microbiol. (2005) 40:274–7. 10.1111/j.1472-765X.2005.01676.x 15752217

[76] Goyal AK Zylstra GJ. Genetics of naphthalene and phenanthrene degradation by *Comamonas testosteroni*. J Ind Microbiol Biotechnol. (1997) 19:401–7. 10.1038/sj.jim.2900476 9451837

[77] Igwaran A Iweriebor BC Ofuzim Okoh S Nwodo UU Obi LC Okoh AI. Chemical constituents, antibacterial and antioxidant properties of the essential oil flower of *Tagetes minuta* grown in Cala community Eastern Cape, South Africa. BMC Complement Altern Med. (2017) 17:351. 10.1186/s12906-017-1861-6 28676058 PMC5497343

[78] Disner GR Calado SL Assis CS Cestari MM. Toxicity of naphthalene in the neotropical fish *Astyanax lacustris* (Characiformes: Characidae) and *Geophagus brasiliensis* (Perciformes: Cichlidae). Evidência. (2017) 17:e12976. 10.18593/eba.v17i1.12976

[79] Fialová SB Rendekova K Mucaji P Slobodnikova L. Plant natural agents: polyphenols, alkaloids and essential oils as perspective solution of microbial resistance. Curr Organ Chem. (2017) 21:1875–84. 10.2174/1385272821666170127161321

[80] Daferera DJ Tarantilis PA Polissiou MG. Characterization of essential oils from *lamiaceae* species by fourier transform Raman spectroscopy. J Agric Food Chem. (2002) 50:5503–7. 10.1021/jf0203489 12236670

[81] Dziri S Casabianca H Hanchi B Hosni K. Composition of garlic essential oil (*Allium sativum* L.) as influenced by drying method. J Essent Oil Res. (2014) 26:91–6. 10.1080/10412905.2013.868329

[82] Leimkühler S Bühning M Beilschmidt L. Shared sulfur mobilization routes for tRNA thiolation and molybdenum cofactor biosynthesis in prokaryotes and eukaryotes. Biomolecules. (2017) 7:5. 10.3390/biom7010005 28098827 PMC5372717

[83] Magariños B Romalde JL Santos Y Casal JF Barja JL Toranzo AE. Vaccination trials on gilthead seabream (*Sparus aurata*) against *Pasteurella piscicida*. Aquaculture. (1994) 120:201–8. 10.1016/0044-8486(94)90078-7

[84] Pujalte MJ Sitjà-Bobadilla A Álvarez-Pellitero P Garay E. Carriage of potentially fish-pathogenic bacteria in *Sparus aurata* cultured in Mediterranean fish farms. Dis Aquat Organ. (2003) 54:119–26. 10.3354/dao054119 12747637

[85] Silva FC Nicoli JR Zambonino-Infante JL Kaushik S Gatesoupe FJ. Influence of the diet on the microbial diversity of faecal and gastrointestinal contents in gilthead sea bream (*Sparus aurata*) and intestinal contents in goldfish (*Carassius auratus*). FEMS Microbiol Ecol. (2011) 78:285–96. 10.1111/j.1574-6941.2011.01155.x 21692817

[86] Itoi S Okamura T Koyama Y Sugita H. Chitinolytic bacteria in the intestinal tract of Japanese coastal fishes. Can J Microbiol. (2006) 52:1158–63. 10.1139/w06-082 17473885

[87] Moi IM Roslan NN Leow ATC Ali MSM Rahman RNZRA Rahimpour A. The biology and the importance of *Photobacterium* species. Appl Microbiol Biotechnol. (2017) 101:4371–85. 10.1007/s00253-017-8300-y 28497204

[88] Rico RM Tejedor-Junco MT Tapia-Paniagua ST Alarcón FJ Mancera JM López-Figueroa F. Influence of the dietary inclusion of *Gracilaria cornea* and *Ulva rigida* on the biodiversity of the intestinal microbiota of *Sparus aurata* juveniles. Aquac Int. (2016) 24:965–84. 10.1007/s10499-015-9964-x

[89] Beltrán JMG Silvera DG Ruiz CE Campo V Chupani L Faggio C. Effects of dietary *Origanum vulgare* on gilthead seabream (*Sparus aurata* L.) immune and antioxidant status. Fish Shellfish Immunol. (2020) 99:452–61. 10.1016/j.fsi.2020.02.040 32084538

[90] Hernandez LH Hardy RW. Vitamin A functions and requirements in fish. Aquac Res. (2020) 51:3061–71. 10.1111/are.14667

[91] Filocamo A Nueno-Palop C Bisignano C Mandalari G Narbad A. Effect of garlic powder on the growth of commensal bacteria from the gastrointestinal tract. Phytomedicine. (2012) 19:707–11. 10.1016/j.phymed.2012.02.018 22480662

[92] Yin D Du E Yuan J Gao J Wang Y Aggrey SE. Supplemental thymol and carvacrol increases ileum *Lactobacillus* population and reduces effect of necrotic enteritis caused by *Clostridium perfringes* in chickens. Sci Rep. (2017) 7:7334. 10.1038/s41598-017-07420-4 28779076 PMC5544757

[93] Laity J Lee B Wright P. Zinc finger proteins: new insights into structural and functional diversity. Curr Opin Struct Biol. (2001) 11:39–46. 10.1016/S0959-440X(00)00167-6 11179890

[94] Will CL Lührmann R. Spliceosomal UsnRNP biogenesis, structure and function. Curr Opin Cell Biol. (2001) 13:290–301. 10.1016/S0955-0674(00)00211-8 11343899

[95] Zeng H Nanayakkara GK Shao Y Fu H Sun Y Cueto R. DNA checkpoint and repair factors are nuclear sensors for intracellular organelle stresses—inflammations and cancers can have high genomic risks. Front Physiol. (2018) 9:516. 10.3389/fphys.2018.00516 29867559 PMC5958474

[96] Wang J An H Mayo MW Baldwin AS Yarbrough WG. LZAP, a putative tumor suppressor, selectively inhibits NF-kappaB. Cancer Cell. (2007) 12:239–51. 10.1016/j.ccr.2007.07.002 17785205

[97] Nakad R Schumacher B. DNA damage response and immune defense: links and mechanisms. Front Genet. (2016) 7:147. 10.3389/fgene.2016.00147 27555866 PMC4977279

